# Ex Vivo Spatiotemporal Characterization of Spermatogenesis in Mouse Testicular Organoids

**DOI:** 10.1002/advs.202512670

**Published:** 2025-10-31

**Authors:** Jiachen Sun, Lifa Zhu, Yutong Li, Jinyan Tang, Tao Zhang, Hengjia Zhang, Wanchu Wang, Lufan Li, Shuhui Bian, Xin Wu

**Affiliations:** ^1^ State Key Laboratory of Reproductive Medicine and Offspring Health Nanjing Medical University Nanjing Jiangsu 211166 China

**Keywords:** In vitro spermatogenesis, infertility, meiosis, testicular organoids

## Abstract

Testicular organoids (TOs) offer an opportunity to preserve fertility, but current TO protocols are limited by suboptimal maintenance of the organoid structure, meiotic defects, and incomplete in vitro spermatogenesis (IVS). Here, a strategy is developed for self‐reconstitution of single‐cell suspensions of neonatal mouse testes into TOs containing the major testicular cell types and generation of tubular‐like structures and haploid spermatids. Morphological and lineage‐specific marker analyses revealed that these TOs met the criteria for organoids, including germ cell differentiation and recapitulation of key events in meiosis (e.g., chromosome recombination and synapsis). Notably, the spatiotemporal characteristics of spermatogenesis in the TOs are comparable to those in testes, and their derived haploids resembled step‐4/5‐like round spermatids in vivo. Further scRNA‐seq analysis confirmed that spermatid‐like cells accounted for ≈2.43% of the total germ cells. Next, undifferentiated germ cells are able to develop into haploid spermatids even when chimeric TOs are reconstructed via testicular somatic cells (Sertoli cells or Leydig cells) from mice with different genetic backgrounds. Collectively, the TO‐based findings provide a promising platform for studies on testicular microenvironment construction, IVS, and fertility preservation.

## Graphical abstract

To date, in vitro spermatogenesis remains a challenge in reproductive medicine. In this study, Sun et al. developed a promising in vitro strategy to generate testicular organoids that enable germ cells from newborn mice to enter meiosis, complete chromosome recombination and synapsis, and develop into haploid spermatids. Their study provides the possibility of preserving fertility through in vitro techniques.

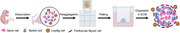

## 1 Introduction

Spermatogenesis is one of the most conserved yet complex cellular developmental processes. In mice, the first wave of spermatogenesis begins just a few days after birth. Continuous spermatogenesis then begins and encompasses three phases: spermatogonial proliferation, spermatocyte meiosis, and spermatid morphological changes. Spermatogonial differentiation occurs within the first few days after birth (5–6 days), followed by the beginning of meiosis (12–14 days), and the appearance of haploid spermatids (≈ 20 days). The entire process takes ≈35 days.^[^ ^1^, ^2^, ^3^ ^]^ In contrast, in vitro spermatogenesis (IVS) is the process of generating functional haploid spermatids from spermatogonial stem cells (SSCs), early‐stage spermatogonia or germline‐competent cells in vitro by simulating the in vivo developmental environment.^[^ ^4^ ^]^ IVS has great potential in the treatment of male infertility (such as that associated with nonobstructive azoospermia), the preservation of fertility in prepubertal cancer patients, and the study of reproductive biology.^[^ ^5^ ^]^ To date, achieving IVS remains a challenge in reproductive medicine.

Previous IVS studies based on rodent or human 2D culture or coculture systems reported that stem cells from multiple sources can differentiate and partially or fully achieve spermatogenesis in vitro, even though some of these development processes significantly differ in terms of the temporal and spatial order of germ cell reprogramming in vivo. These stem cells are derived from prospermatogonia, postnatal SSCs,^[^ ^6^, ^7^ ^]^ embryonic stem cells (ESCs)^[^ ^8^, ^9^ ^]^ or reprogrammed induced pluripotent stem cells (iPSCs).^[^ ^10^, ^11^, ^12^ ^]^ However, most reports show that haploid spermatids generated in vitro on the basis of 2D culture systems have low yields, incomplete lineages, or ethical concerns that must be resolved prior to potential clinical application.

As early as the 1960s, Steinberger et al. explored the in vitro culture conditions for human testicular tissue, including culture medium, pH value, temperature, and oxygen and carbon dioxide tension, and reported that immature germ cells could be maintained in a culture system for several weeks.^[^ ^13^ ^]^ Despite various attempts at optimization in the decades since then, the precise conditions for the optimal in vitro maturation of germ cells in testicular tissue remain unclear. In 2011, Sato et al. developed undifferentiated spermatogonia into mature sperm by culturing mouse testicular tissue organs.^[^ ^14^ ^]^ In addition, in 2020, Yuan et al. cultured human fetal gonads for ≈50 days, reproduced the transformation of spermatogonial precursors to round spermatids in vitro, and reported that haploid cells were competent for fertilization.^[^ ^15^ ^]^ Notably, among the described IVS strategies, in vitro culture of mouse testis fragments is likely the most successful approach to date for supporting the entire process of spermatogenesis.^[^ ^14^, ^16^, ^17^ ^]^

Organoids are 3D cultures that are self‐assembled from single‐cell suspensions from tissues, and their structural features closely resemble the spatial organization and function of cells in the native tissues they mimic.^[^ ^18^ ^]^ Since testicular organoids (TOs) can simulate the structure and function of the testis in vitro, enabling investigations of which cells can be controlled and how they are reorganized (which is not feasible in organotypic culture), the applications of the TO system have been expanded, making it possible to study the germ cell microenvironment, spermatogenesis, testicular development and male infertility, and may eventually achieve the in vitro production of functional sperm.^[^ ^19^ ^]^ Importantly, TOs can be generated from human and large animal testicular biopsies that are inaccessible for in vivo studies, allowing for the modeling of testicular disease or cancer and the investigation of potential therapeutic interventions.^[^ ^20^ ^]^ For example, AbuMadighem et al. used testis chip technology to differentiate germ cells into spermatocytes and haploid spermatids,^[^ ^21^ ^]^ the Dobrinski laboratory has made many pioneering explorations in constructing testicular organoids of mice, rats, pigs, etc.,^[^ ^22^, ^23^, ^24^ ^]^ and Yoni Baert et al. attempted to construct human testicular organoids.^[^ ^25^ ^]^ However, while the potential benefits of TOs have been recognized, attempts to reproduce testicular structure and IVS function remain limited and insufficient.^[^ ^26^ ^]^

In this study, we developed a strategy for self‐reorganization of testicular single‐cell suspensions into TOs that contain major testicular cell types and form seminiferous tubule‐like structures. The nutritional and temperature conditions used for the cultured TOs were based mainly on the testicular tissue culture system.^[^ ^14^ ^]^ The TOs we constructed met the criteria for organoids, and importantly, the spermatogonia from the testes of newborn 6‐day‐old mice were able to be maintained and successfully enter meiosis; complete chromosome recombination and synapsis; and develop into step‐4/5 round spermatid‐like haploids in vitro.

## 2 Results

### 2.1 Testicular Organ Culture Supports Mouse Spermatogenesis, with a Spatiotemporal Order Similar to that Observed In Vivo

**Figure 1:**
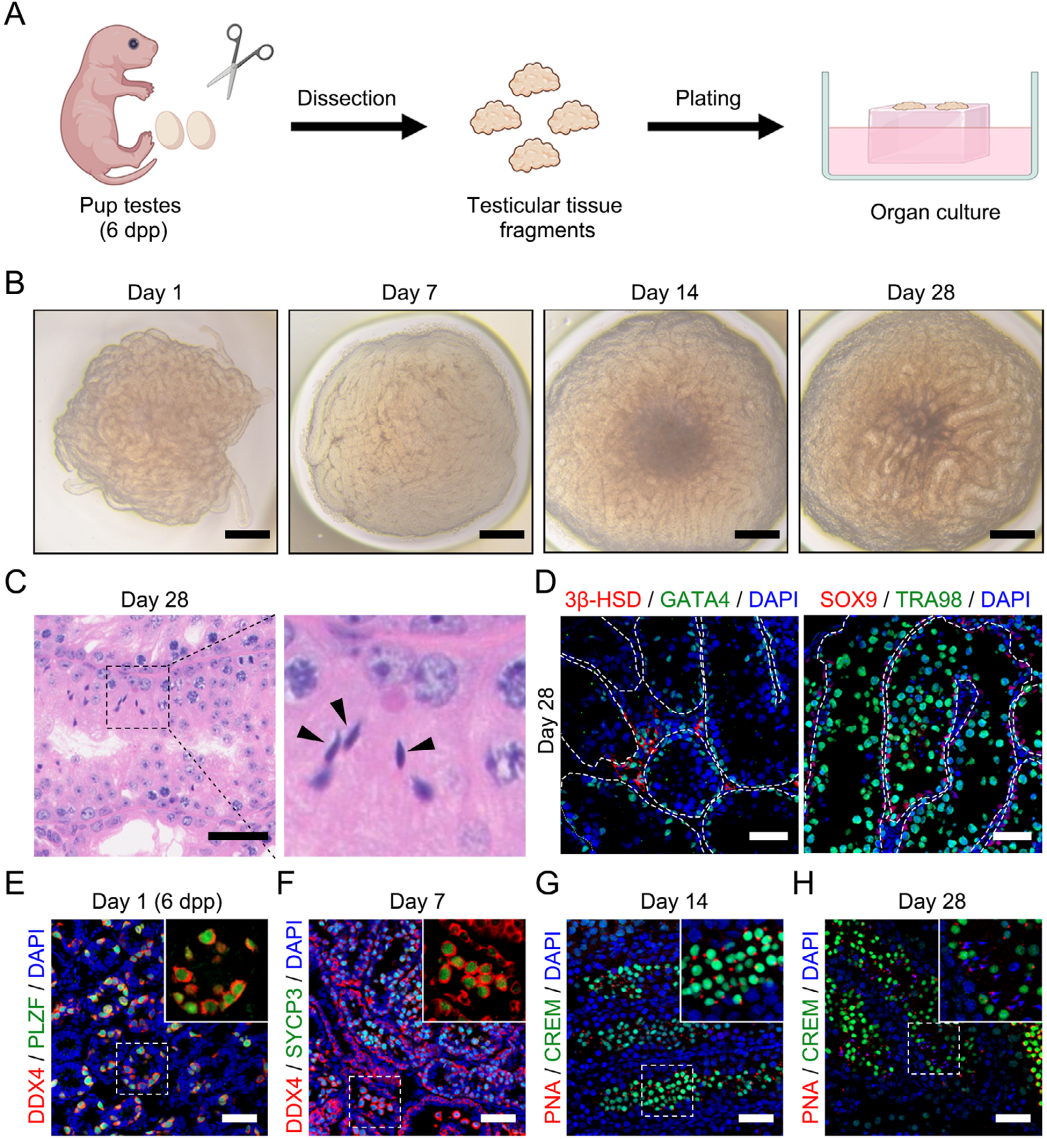
In vitro spermatogenesis using organ culture. A) Schematic illustration of the organ culture protocol. Testes were dissected from 6‐day postpartum (dpp) mouse pups, cut into small tissue fragments, and cultured at the air‒liquid interface. B) Representative bright‐field images of OCs (testicular tissue) cultured on days 1, 7, 14, and 28. Scale bar, 100 µm. C) Hematoxylin‒eosin (H&E) staining of OC fragments cultured for 28 days. The magnified inset (right panel) highlights the presence of elongated spermatids (black arrowheads), characterized by their condensed and hook‐shaped nuclei. Scale bar, 50 µm. D) Immunostaining of OCs cultured for 28 days shows testicular architecture and cell distribution, with costaining of the Leydig cell marker 3β‐HSD (red; left) and the somatic cell marker GATA4 (green; left), and costaining of the Sertoli cell marker SOX9 (red; right) and the germ cell marker TRA98 (green; right). DAPI (blue) was used to stain the cell nuclei. The white dashed lines outline the seminiferous tubules. Scale bar, 50 µm. E–H) Immunofluorescence analysis of cultured tissue tracking the temporal progression of spermatogenesis using stage‐specific markers. Insets show higher magnification images of the boxed regions. On day 1 (6 dpp mouse testis), the tubules contain a population of DDX4‐positive (red) germ cells, many of which are colabeled with PLZF (green), a marker for undifferentiated spermatogonia (E). Immunostaining for DDX4 (red) and SYCP3 (meiotic marker, green) in cultured tissue on day 7, showing the presence of spermatocytes (F). Immunostaining for peanut agglutinin (PNA, an acrosomal marker, red) and CREM (postmeiotic germ cell marker, green) in cultured tissue on day 14, revealing the presence of round spermatids (PNA^+^/CREM^+^) (G) Immunostaining for PNA (red) and CREM (green) in cultured tissue on day 28, showing the presence of round spermatids and elongated spermatids (PNA^+^/CREM^−^). Nuclei were counterstained with DAPI (blue) (H). Scale bar, 50 µm.

The first step in achieving in vitro spermatogenesis is to simulate the microenvironment effectively, including structural support, nutritional support, and signal transduction.^[^ ^4^ ^]^ To explore the prerequisites for spermatogenesis in vitro, we first reproduced the mouse testis organ cultures (OCs) established by Sato et al.^[^ ^14^ ^]^ We processed mouse testis tissue from 6‐day‐old newborn pups into fragments ≈1–3 mm in diameter, placed them on agarose blocks half‐submerged in organ culture medium (**Figure** **1**A; Tables S1 and S2, Supporting Information for details), and cultured them at 34 °C for 4 weeks. The seminiferous tubule‐like structure in the cultured tissue was maintained throughout the 28‐day culture period (bright field, Figure 1B). In tissues cultured for 28 days, we were able to observe spermatogenic cells at different stages, and importantly, elongated spermatids with condensed nuclei were observed in tubules (Figure 1C). In addition, by co‐staining the testicular somatic cell marker GATA4 and the Leydig cell marker 3β‐HSD, we found that the localization of the testicular somatic cell population was consistent with that in vivo, while by co‐staining the testicular Sertoli cell marker SOX9 and the germ cell marker TRA98, we found that the distribution of the germ cell population after 28 days of in vitro culture was the same as that in mouse testes (Figure 1D; Figure S1A, Supporting Information).

Next, we observed the spatiotemporal status of germ cell development on day 1 (when the tissue had just started to be cultured), day 7, day 14, and day 28 of OCs (the time points correspond to the approximate timing of spermatogonial differentiation, meiosis, and the formation of round and elongated spermatids during the first wave of spermatogenesis in vivo)^[^ ^3^ ^]^ by costaining the germ cell marker DDX4 with the undifferentiated spermatogonia marker PLZF, the meiotic cell marker SYCP3, the secondary spermatocyte to early round spermatid marker CREM (cAMP response element modulator), and the sperm acrosome marker PNA (peanut agglutinin). As expected, most germ cells of the newly cultured tissues were PLZF‐positive undifferentiated spermatogonia (Figure 1E), while DDX4‐ and SYCP3‐positive meiotic germ cells appeared in the seminiferous tubules after 7 days of culture (Figure 1F), CREM‐ and PNA‐positive round spermatids were found after 14 days of culture (Figure 1G), and PNA‐positive, CREM‐negative elongated spermatids were found after 28 days of culture (Figure 1H). This result shows that although *ex vivo*, testes are not precisely regulated by the microenvironment in the body, the time of each stage of their germ cell development is comparable to that in vivo.^[^ ^3^ ^]^ Notably, the timing of germ cell development in cultured neonatal tissues corresponds to the timing of spermatogenesis in vivo, providing a reference framework for subsequent spatiotemporal analysis of spermatogenesis in vitro in testicular organoids.

### 2.2 Establishment of an In Vitro Mouse TO Culture Strategy and Generation of Haploid Spermatids

**Figure 2:**
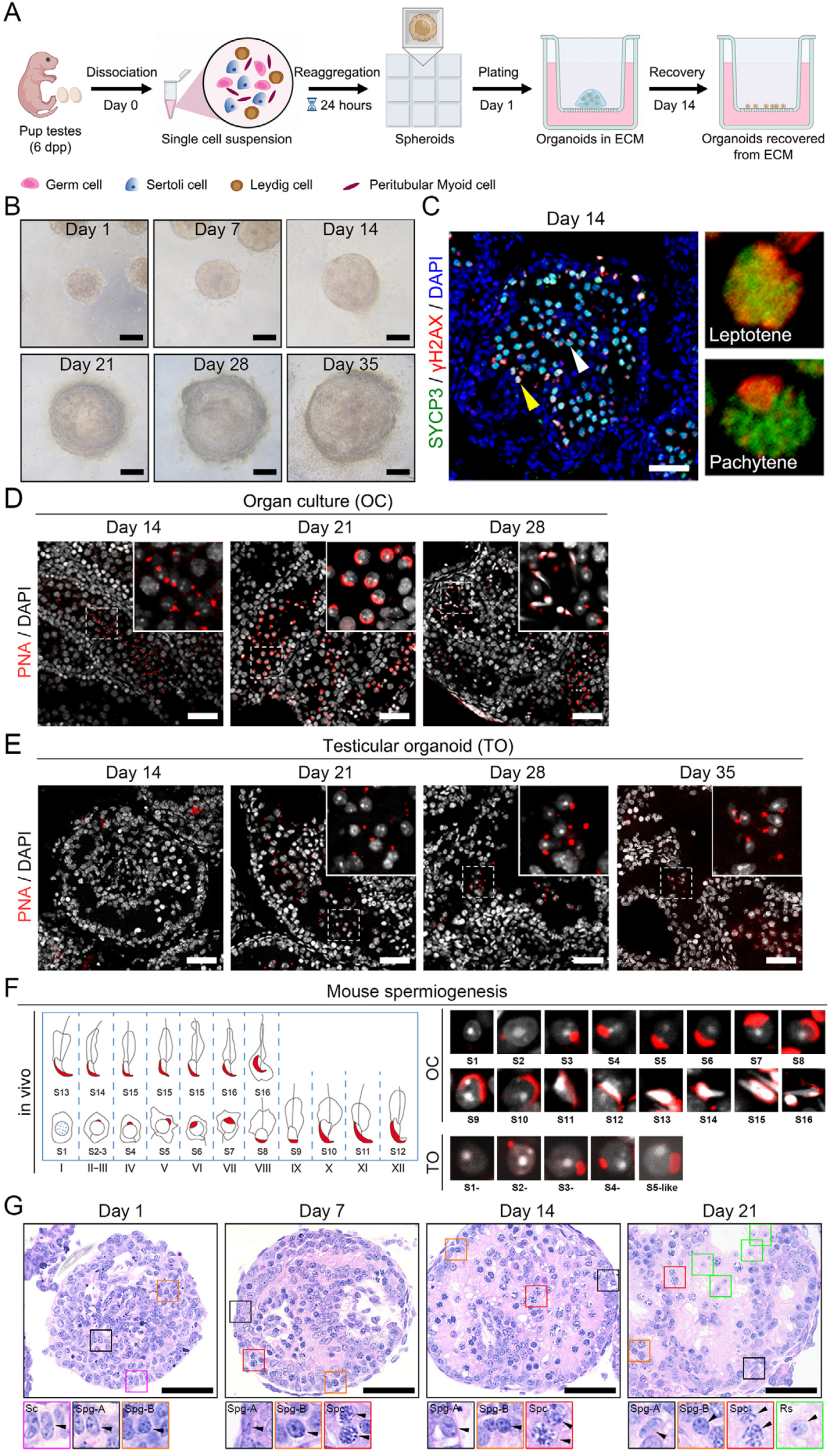
In vitro spermatogenesis in TOs. A) Schematic diagram of the TO culture preparation procedure. B) Representative bright field images of organoids based on 4000 cells/spheroid size at 1, 7, 14, 21, 28, and 35 days in culture. Scale bar, 100 µm. C) Immunofluorescence staining of 14‐day organoid sections showing representative meiotic progression. DAPI (blue), SYCP3 (green), and γH2AX (red) are shown. The yellow arrow indicate leptotene spermatocytes, and the white arrow tips point to pachytene spermatocytes. Scale bar, 50 µm. D) Immunostaining of OCs on the indicated culture days. PNA (red) and DAPI (white) were used to identify haploid spermatids. Scale bar, 50 µm. E) Immunostaining of TOs on the indicated culture days. PNA (red) and DAPI (white) were used to identify haploid spermatids. Scale bar, 50 µm. F) A schematic diagram of spermatogenesis in mice was used as a control to compare the spermiogenesis achieved by OCs and TOs, and PNA fluorescence‐positive labeling of the acrosome. G) H&E staining of organoid sections on day 1, 7, 14 and 21 of culture. Sertoli cells (Sc, purple box), type A spermatogonia (Spg‐A, black box), type B spermatogonia (Spg‐B, orange box), spermatocytes (Spc, red box), and round spermatids (Rs, green box) are indicated in the representative organoid sections. Scale bar, 50 µm.

Next, we developed a 3D culture‐based strategy for the reconstruction and in vitro culture of mouse TOs. This 3D culture system is designed to recapitulate the architecture of the testis, providing a microenvironment conducive to the complex intercellular and cell‐matrix interactions known to be critical for spermatogenesis.^[^ ^27^ ^]^ Unlike organ cultures, the TOs were derived from the reconstruction of single cells from mouse testes (**Figure** **2**A). Briefly, we digested and filtered single‐cell suspensions of testis tissue from mice at postnatal day 6, and the cells were seeded into the microwells of a CA600 high‐throughput 3D Spheroid Preparation Kit to form cell spheroids (≈4000 cells per spheroid) and cultured for 24 h. After 24 h, the cell spheroids were collected with a pipette and resuspended in commercial extracellular matrix (ECM) reagent (Basement Membrane Extract, BME). Then, ≈20 µL of the solution was added dropwise to the cell culture insert in a 12‐well cell culture plate. After the ECM solidified for half an hour, ≈0.5 mL of culture medium was added to the outer chamber of each well of a 12‐well culture plate equipped with an insert and cultured at 34 °C. In contrast, some organoids collapse without an initial ECM embedding. After 14 days of culture, we removed the ECM of the TOs by using a cell recovery solution and then seeded the ECM‐free TOs directly into the insert of the plate for further culture (see Methods for a more detailed description) (Figure 2A). TO cultured in 10% KnockOut serum replacement (KSR) medium supplemented with hormones and antioxidants was greatly improved compared with simple OC medium (Figure S2A; Table S3, Supporting Information). In addition, compared with the TOs that formed immediately after aggregation (day 1), the TOs cultured for 7 days had smooth and clear edges, and the edge structure was translucent and radial. We then found that after 14 days of culture, the edge of the TO became thicker, and although the interior still maintained a tubular structure, the center of the TO began to darken (Figure 2B). By removing the ECM and reseeding to eliminate the potentially deleterious effects of long‐term embedding (Figure 2A; Figure S2B, Supporting Information), we were able to maintain TOs in vitro for over 35 days (Figure 2B).

Next, we aimed to determine whether the spermatogonia from the TOs of 6‐day‐old mice could differentiate and continue developing. Immunostaining of TOs cultured for 14 days revealed the presence of spermatocytes in tubules. These spermatocytes were double positive for the synaptonemal complex marker SYCP3 and the DNA double‐strand break marker γH2AX, indicating that the germ cells in the TOs could develop to the meiotic stage. The nuclear localization of γH2AX further indicated that the germ cells developed at least to the pachytene stage of primary spermatocytes (Figure 2C).

After carefully evaluating the development of mouse spermatids in mouse OCs, we then investigated whether haploid spermatids could form in the TOs. Compared with OCs, spermatogonia isolated from 6‐day‐old mice developed into haploid spermatids after 21 days of culture in reconstructed TOs, although it took ≈1 week longer than observed for OCs because the single cells needed to be reconstituted into a tubule‐like structure (Figure 2D,E). Immunostaining of mouse TOs revealed PNA‐positive cells with weak dot‐like signals in the tubules after 21 days of culture (Figure 2E). Compared with the characteristic acrosome formation stages observed in vivo (Figure 2F),^[^ ^28^ ^]^ these cells in TOs presented morphological characteristics typical of step 1–2 round spermatids, and on day 28 and day 35 (Figure 2E), we identified only step‐4/5‐like round spermatids, in which the acrosome structure began to extend from the anterior pole of the nucleus to the equatorial node. Compared with organoids cultured on days 1, 7, and 14, morphological staining of TO sections on day 21 further confirmed the presence of spermatogonia, spermatocytes, and round spermatid‐like haploid cells in TO tubules (Figure 2G; day 28 testes served as the in vivo morphological control, Figure S1A, Supporting Information). However, compared with organ cultures (Figure 2F), in which we were able to identify round spermatids in all developmental steps consistent with in vivo development by PNA‐specific acrosome staining (Figure 2F, steps 1–16), in the subsequent TO culture, although we adjusted many culture conditions, we were unable to observe more mature round spermatids after step 5, indicating that our constructed mouse TO culture system was insufficient to support complete spermiogenesis (Figure 2F). Nonetheless, these data indicate that our constructed TO culture system can support spermatogenesis in vitro and that undifferentiated spermatogonia in postnatal mouse TOs can develop into step‐4/5 haploid round spermatids in vitro.

### 2.3 Functional Assessment and Characterization of Mouse TOs

**Figure 3:**
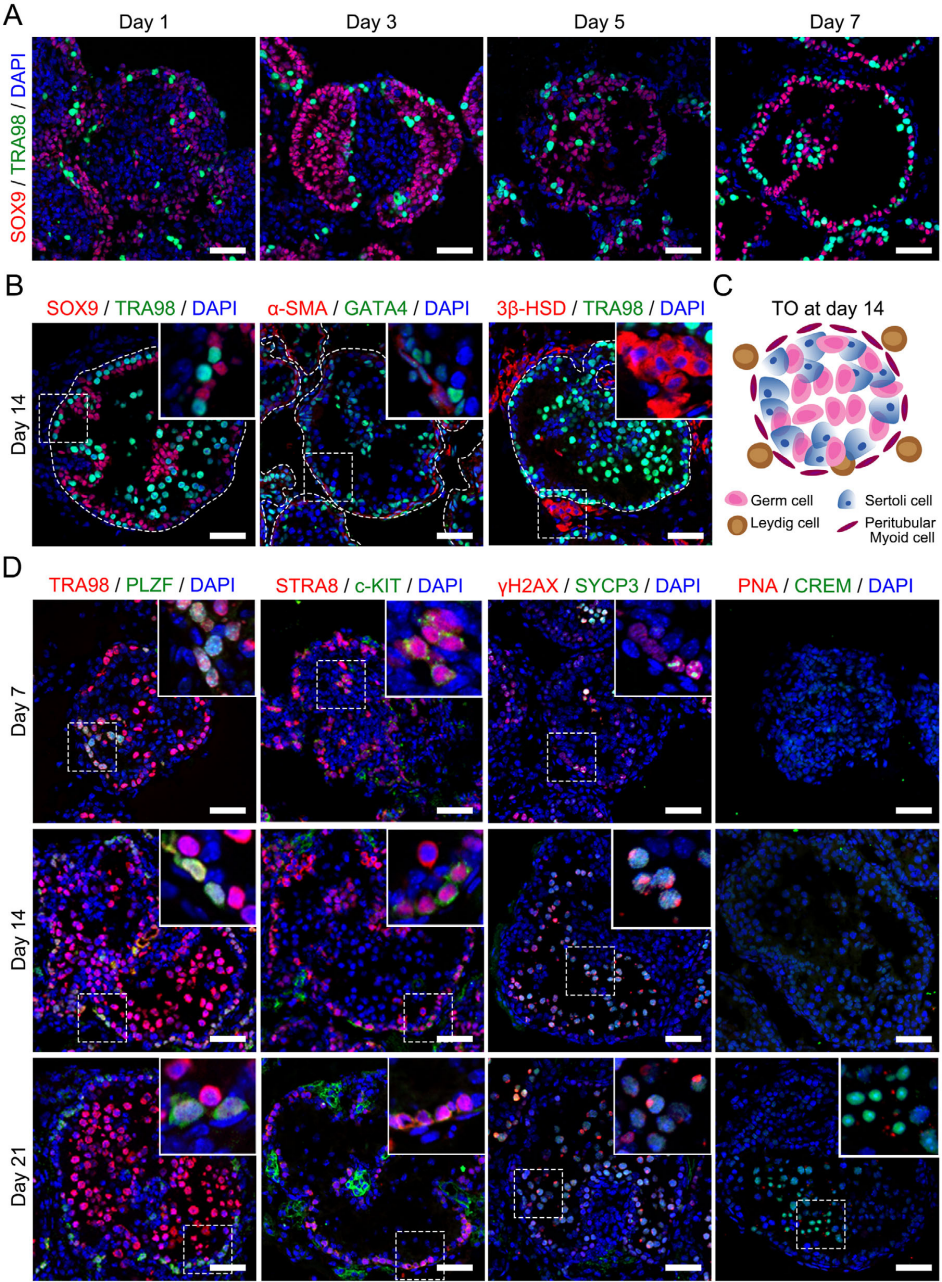
Functional assessment and characterization of mouse TOs. A) Time‐course immunofluorescence analysis of the self‐organization of testicular cells into organoids during the first week. The dynamic process of testicular cell rearrangement to form a tubule‐like structure can be observed by staining Sertoli cells with SOX9 and germ cells with TRA98. Scale bar, 50 µm. B) Identification of major testicular cell types in the organoids on day 14 of culture. Immunostaining for SOX9 (Sertoli cells), TRA98 (germ cells), α‐SMA (peritubular myoid cells), GATA4 (somatic cells) and 3β‐HSD (Leydig cells) was conducted. Seminiferous tubules are outlined by white dashed lines. Nuclei were stained with DAPI (blue). Scale bar, 50 µm. C) Distribution and localization of mouse testicular cells in TOs after 14 days of culture. D) Immunofluorescence staining on day 7 (upper panel), 14 (middle panel), and 21 (lower panel) of TO culture revealed undifferentiated spermatogonia that colocalized with the germ cell marker TRA98 and were PLZF positive; differentiated spermatogonia or early meiotic cells that were c‐KIT and STRA8 positive; meiotic cells that were γH2AX and SYCP3 positive; and postmeiotic cells that were CREM and acrosomal PNA positive. Scale bar, 50 µm.

Given that in vitro TOs need to meet at least three criteria, namely, they must contain multiple cell types that are specific to an organ, be able to self‐reorganize into structures that approximate the organ they mimic, and be able to recapitulate certain in vivo organ‐specific functions,^[^ ^18^ ^]^ we next assessed whether the reconstructed mouse TOs met these criteria. First, we tracked the distribution dynamics of individual cell types in TOs generated from testes isolated from six‐day‐old mice by tracking cells positive for the Sertoli cell marker SOX9 and the germ cell marker TRA98 (**Figure** **3**A). We found that on day 1 of culture (before ECM embedding), these cells were randomly dispersed in the spheroids, whereas on days 3 and 5, Sertoli cells (SCs) began to envelop the germ cells, and by day 7, most SCs had migrated to the tubular basement membrane. The migration pattern of SCs during remodeling may mimic early testicular cord formation in vivo.^[^ ^29^ ^]^ Next, we characterized the TO architecture after 14 days of culture using the somatic cell marker GATA4, the myoid cell marker α‐SMA, the interstitial Leydig cell marker 3β‐HSD, and the germ cell marker TRA98. The results revealed the presence of compartmentalized tubular structures within the TOs, similar to in vivo testicular tissue (Figure 3B; Figure S1A, Supporting Information). GATA4‐positive somatic cells and α‐SMA‐positive peritubular myoid cells are located at the periphery of the tubular structure, 3β‐HSD‐positive Leydig cells are located in the interstitial area between the tubules, and germ cells are located in the tubules (Figure 3B,C).

Next, we investigated whether spermatogenesis in TOs also mimics the spatiotemporal characteristics of the testis in vivo. We used the undifferentiated spermatogonia marker PLZF, the differentiated spermatogonia marker c‐KIT, the early meiotic marker STRA8, the spermatocyte markers SYCP3 and γH2AX, the late meiotic phase marker CREM, and the sperm acrosome marker PNA to evaluate the developmental characteristics of germ cells in TOs at different time points. In addition, we also observed that PLZF‐positive undifferentiated spermatogonia were localized at the peripheral area of the tubular structure throughout the culture period (Figure 3D), and we found that c‐KIT‐positive differentiated spermatogonia and STRA8‐positive early meiotic cells were detectable on the 7th day of culture and at later time points. Moreover, few leptotene or zygotene SYCP3 and γH2AX double‐positive spermatocytes were observed in the tubules after 7 days of culture, while pachytene spermatocytes were observed after 14 days of culture (Figure 3D), and PNA and CREM double‐positive spermatids were observed after 21 days of culture (Figure 3D). Quantitative immunofluorescence analysis revealed that the ratio of total TRA98‐positive germ cells to SOX9‐positive SCs remained consistent over the 21‐day culture period (Figure S3A, Supporting Information), and the proportion of PLZF‐positive undifferentiated spermatogonia decreased after 7 days of culture but remained consistent after 14 and 21 days (Figure S3B, Supporting Information). From day 7 to day 21 of culture, SYCP3‐positive germ cells entering meiosis could be observed in the TOs, and their proportion remained relatively consistent during the culture period (Figure S3C, Supporting Information). Most importantly, PNA and CREM double‐positive round spermatid‐like cells were detected on day 21, and tubules containing these double‐positive cells (even if there were only a few spermatid‐like cells) accounted for 61.36% ± 21.24% of the total tubules (Figure S3D, Supporting Information). The formation of tight junctions between adjacent SCs is crucial for establishment of the blood‒testis barrier (BTB) and the spermatogenic microenvironment in vivo. We next performed immunofluorescence staining for the tight junction proteins CLDN11 (Claudin‐11) and ZO‐1 (Zonula Occludens‐1) in TOs on day 14 of culture (Figure S3E,F, Supporting Information) and detected continuous CLDN11‐ and ZO‐1‐positive tight junctions between GATA4‐positive SCs, which distinguished the basal and apical compartments to support spermatogenic cell development. In addition, we showed by Evans blue staining that fluorescence was confined to the peripheral region of tubular structures in TOs at day 21 of culture (Figure S3G,H, Supporting Information), which is consistent with the staining of seminiferous tubules in vivo, suggesting that BTB function may be established in organoids.

Next, we evaluated the development of somatic cells in mouse TOs. Staining for the proliferation marker KI67 revealed a significant decrease in SCs proliferative activity, indicating that SCs in TOs had matured. In contrast to the low abundance of androgen receptor (AR) signals in 6‐day‐old mouse SCs (GATA4‐positive, oval or elongated nuclei), SCs in 14‐day‐old TOs exhibited detectable AR signals (Figure S4A,B, Supporting Information). We further used quantitative reverse transcription‒PCR (qRT‒PCR) to verify that the expression of anti‐Mullerian hormone (*Amh*) and *Ck18* gradually decreased during TO culture, whereas the transcription level of *Ar* increased (Figure S4C–E, Supporting Information). Taken together, these data indicate that the mouse TOs established in this study meet the basic criteria for organoids, including self‐reorganization into major testicular cell types, formation of seminiferous tubule‐like structures in vivo, and support for germ cell development.

### 2.4 scRNA‐seq Analysis Reveals the Developmental Trajectory of Germ Cells in Mouse TOs

**Figure 4:**
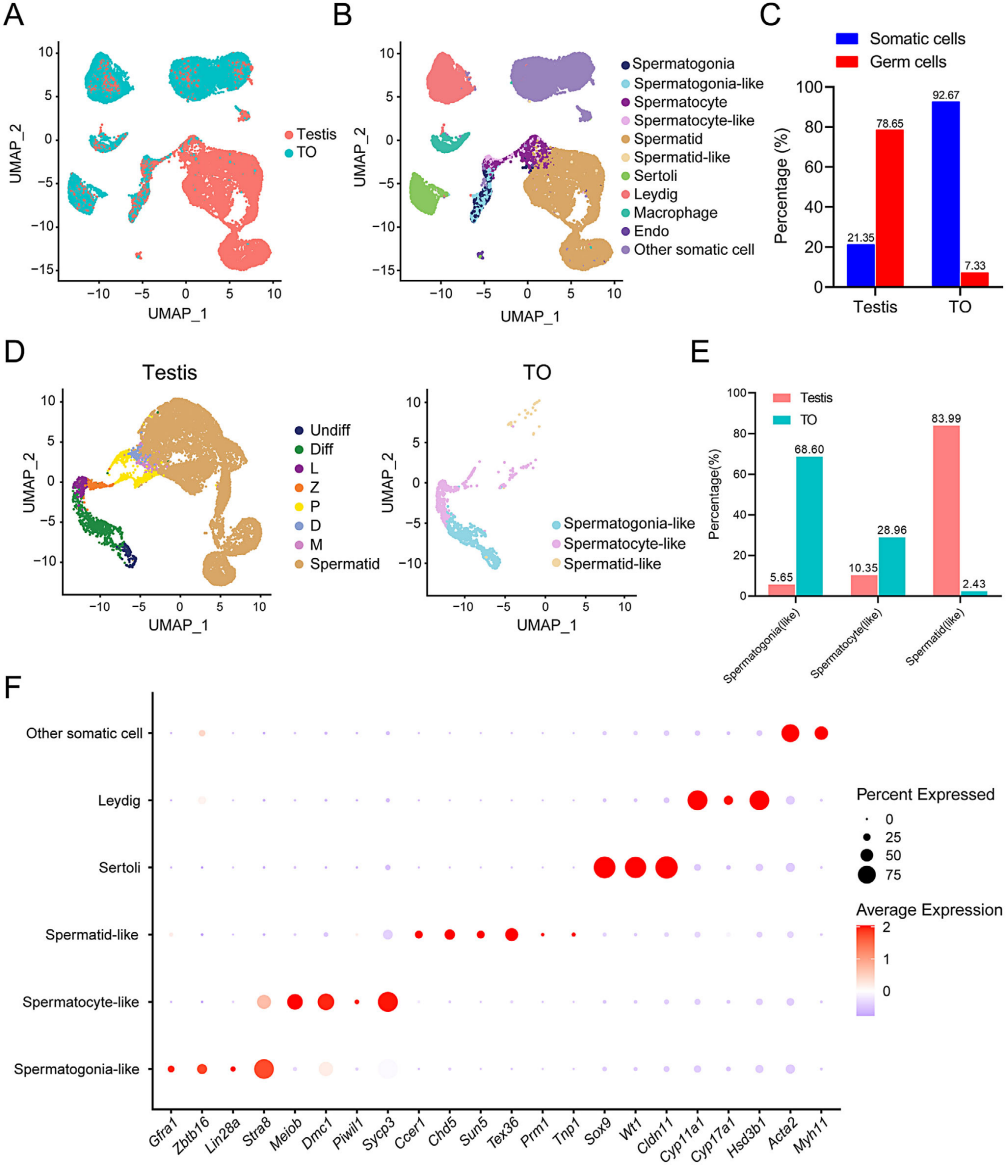
Single‐cell RNA sequencing analysis of mouse TOs. A) UMAP visualization of combined single‐cell transcriptomic data from mouse testes and TOs. Each dot represents a single cell, colored by sample origin. B) UMAP plot showing cell type identification within the combined dataset. The distinct clusters represent different cell types, including spermatogonia(like), spermatocytes(like), spermatids(like), Sertoli cells, Leydig cells, macrophages, endothelial cells (Endo) and other somatic cells. C) Bar plot showing the percentage of somatic cells (blue) and germ cells (red) in the testis and TO samples. D) UMAP projections of single‐cell transcriptomes separated by sample: testis (left) and TO (right). The cells are colored according to their differentiation stage within the germ cell lineage: undifferentiated spermatogonia (Undiff), differentiating spermatogonia (Diff), leptotene spermatocytes (L), zygotene spermatocytes (Z), pachytene spermatocytes (P), diplotene spermatocytes (D), meiotic spermatocytes (M), spermatids, spermatogonia‐like cells, spermatocyte‐like cells, spermatid‐like cells. E) Bar plot showing the percentages of germ cell types in the testis (left) and TO (right) samples. F) Dot plot illustrating the expression of canonical marker genes across the major cell lineages in TOs. The dot size corresponds to the percentage of cells within a cluster expressing the gene, while the color intensity represents the average expression level, and the expression patterns of spermatogonial (*Zbtb16*, *Gfra1*), meiotic (*Meiob*, *Sycp3*, *Dmc1*), postmeiotic (*Ccer1*, *Chd5*, *Sun5*, *Tex36*, *Prm1*, *Tnp1*), Sertoli cell (*Sox9*, *Wt1*, *Cldn11*), Leydig cell (*Cyp11a1*, *Hsd3b1*), and other somatic cell (*Acta2*, *Myh11*) markers validate the accuracy of the cell type annotation.

Next, we performed single‐cell RNA sequencing (scRNA‐seq) of 28‐day‐old TOs and compared the data with single‐cell RNA sequencing data from 28‐day‐old mouse testes to explore cellular development in the TOs and the heterogeneity and composition of the population. We collected a total of 27134 TO cells and 24005 testicular tissue cells for transcriptome analysis using the 10x Genomics Chromium sequencing platform (China). After data quality control (see the Methods for details), 18565 TO cells and 18816 testicular cells were obtained for subsequent analysis. On average, 7446 unique molecular identifiers (UMIs) and 2450 genes were detected per cell, sufficient to characterize different cell types in the mouse testis (Figure S5A–C, Supporting Information). Bioinformatic analysis employing unsupervised clustering revealed eight major clusters across all sequenced testis cells (**Figure** **4**A), and on the basis of the expression patterns of known marker genes in the mouse testis (Figure S5D,E, Supporting Information), we initially identified three germ cell populations (spermatogonia, spermatocytes, and spermatids) and five somatic cell populations (SCs, Leydig cells, macrophages, endothelial cells and other somatic cells) (Figure 4B, cell type identification see the Methods for details). We then performed a statistical analysis of all sequenced cells to determine the proportions of germ cells and somatic cells in TOs and mouse testes. The results revealed that in TOs, germ cells accounted for 7.33% of cells, and somatic cells accounted for 92.67% of cells. In contrast, in mouse testis tissue, germ cells accounted for 78.65% of cells, and somatic cells accounted for 21.35% of cells. This finding suggests that, unlike in vivo, the somatic cell population in TOs is more dominant than the germ cell population and eventually becomes the majority in TOs (Figure 4C).

Nevertheless, we were able to recluster 1360 TO‐derived germ cells and 14799 testis tissue‐derived germ cells to investigate the transcriptomic signatures of germ cell development. Through unsupervised clustering and marker gene identification, we observed 8 distinct germ cell subtypes/states, and despite significant quantitative differences, overall, the order of germ cell development in the TOs in vitro was consistent with that in the testes (Figure 4D). Next, statistical analysis of the germ cell composition revealed that spermatid‐like cells accounted for ≈2.43% of the total germ cells in TOs and 83.99% in testis tissue (Figure 4E). Next, we found that the gene expression patterns in TOs were consistent with expectations, including those genes *Zbtb16* and *Gfrα1* that are specifically expressed in undifferentiated spermatogonia, *Meiob*, *Dmc1*, and *Sycp3* that are specifically expressed in spermatocytes, and known haploid spermatids marker genes *Tnp1* and *Prm1*, as well as newly reported haploid spermatids marker genes including *Ccer1* and *Chd5* ^[^ ^30^, ^31^ ^]^ (Figure 4F).

To explore the differences in the transcriptomes of testicular somatic cells in vivo and in vitro, we performed differential gene expression analysis of the same type of somatic cells in the testes and TOs. We first removed genes that might cause contamination from the differentially expressed genes between the two groups of somatic cells and then selected genes with |log2FC|> 1 and *P*< 0.01 as statistically significant differentially expressed genes. Next, differential gene expression analysis revealed that although most genes did not significantly differ among the various somatic cell types, the analysis of the three somatic cell types (Figure S5F, Supporting Information) revealed that the number of downregulated differentially expressed genes was greater than the number of upregulated differentially expressed genes. Among the cell types, the proportion of downregulated genes in macrophages was the highest (≈14.74%). In contrast, the proportion of downregulated genes in Sertoli cells and Leydig cells was relatively low. We subsequently performed Kyoto Encyclopedia of Genes and Genomes (KEGG) pathway enrichment analysis of the identified DEGs (Figure S5G, Supporting Information). Notably, some pathways associated with important functions of testis somatic cells exhibited changes in the TOs. For example, the genes whose expression may differ between SCs in vivo and in vitro were enriched mainly in pathways such as tight junctions, actin cytoskeleton regulation, and cell apoptosis. KEGG analysis revealed that the loss of specific functions of somatic cells in vitro may be a factor that causes differences in germ cell development in vivo and in vitro. On the basis of the scRNA‐seq data, although the proportion of germ cells at each developmental stage in the TOs was different from that in the testis in vivo, the development times and spatial trajectories were close to those observed in vivo; that is, germ cells isolated from the testes of 6‐day‐old mice can develop from diploid spermatogonia into tetraploid spermatocyte‐like cells and further form haploid spermatid‐like cells in TOs.

### 2.5 Germ Cells in TOs Recapitulate Key Events during Meiosis

Since only a small proportion of spermatid‐like cells was detected in TOs (2.43% in TOs vs 83.99% in vivo, Figure 4E), we further explored whether the difference in germ cell number in TOs was due to severe defects in germ cell development in TOs. We explored the integrity of meiosis in TOs, a key process for spermatogenesis in vitro.

**Figure 5:**
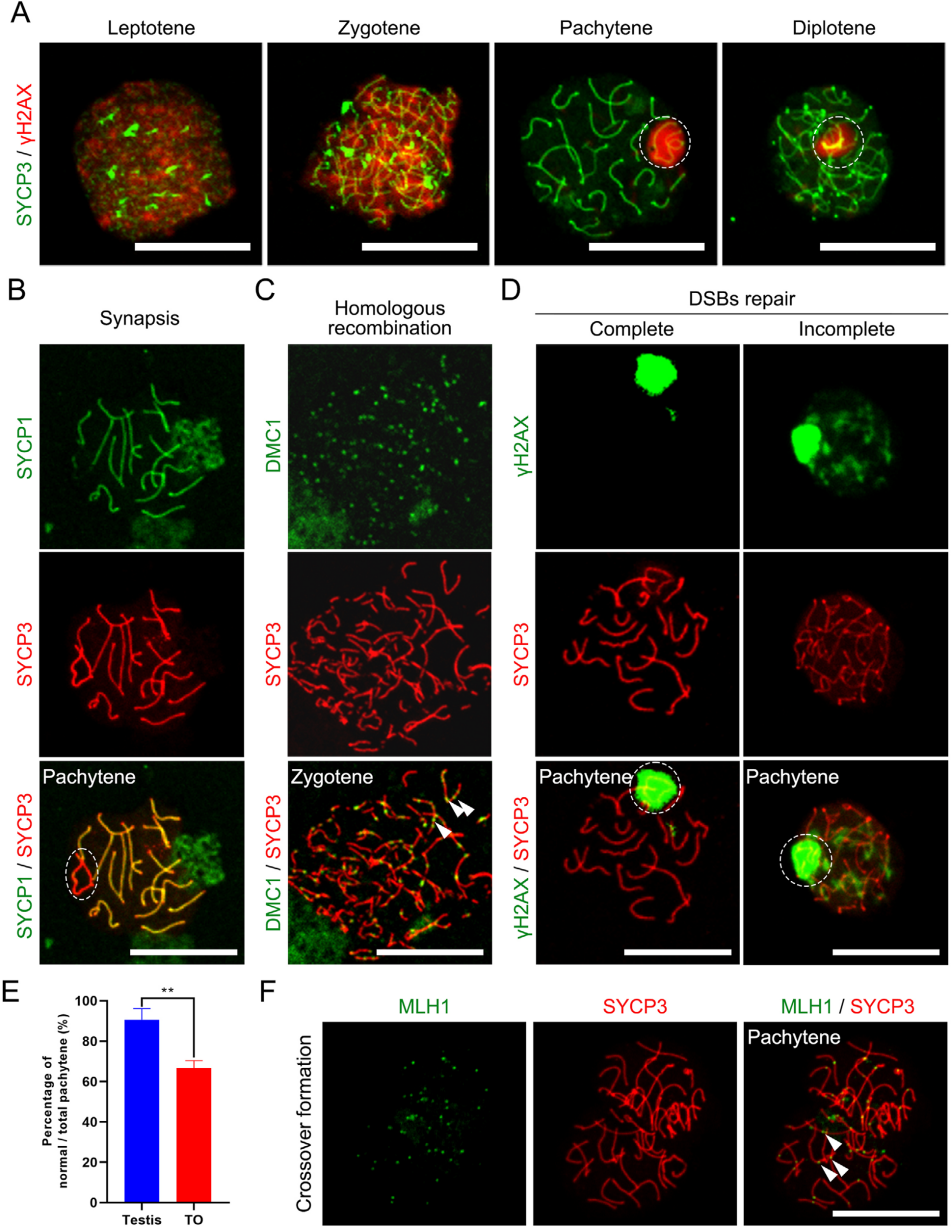
Chromosomal synapsis and recombination during in vitro meiosis. A) Immunofluorescence staining of meiotic spermatocytes in TOs for SYCP3 (green) and γH2AX (red) at different stages of prophase I (leptotene, zygotene, pachytene, and diplotene), showing normal patterns of distribution and colocalization. The dashed circle highlights the sex body. Scale bar, 20 µm. B) Immunofluorescence staining of a pachytene spermatocyte stained for SYCP3 (red) and SYCP1 (green). The extensive colocalization (yellow) of SYCP1 and SYCP3 indicates successful synapsis of homologous chromosomes. The dashed circle highlights the unsynapsed XY chromosome pair (sex body). Scale bar, 20 µm. C) Immunofluorescence staining of a TO‐derived zygotene spermatocyte for SYCP3 (red) and the recombinase DMC1 (green). The presence of numerous DMC1 foci along the SYCP3‐positive chromosome axes confirms the recruitment of the homologous recombination machinery. Scale bar, 20 µm. D) Representative images of pachytene spermatocytes illustrating the criteria for assessing DSBs repair. A “complete” repair state is defined by the γH2AX signal (green) being confined to the sex body (dashed circle), indicating successful repair on autosomes. An “incomplete” state is characterized by persistent γH2AX foci on autosomes in addition to the sex body. Scale bar, 20 µm. E) Quantification of the percentage of normal pachytene spermatocytes (characterized by persistent γH2AX staining) relative to the total number of pachytene spermatocytes in the testis and TOs. The data are presented as the means ± SDs (biologically independent samples, n = 3), ** indicates *P* < 0.01. (F) Immunofluorescence image of a TO‐derived pachytene nucleus costained for SYCP3 (red) and the crossover‐specific marker MLH1 (green). Scale bar, 20 µm.

First, we confirmed the presence of spermatocytes at various stages of meiosis in TOs during 21 days of culture by double staining with γH2AX and SYCP3 antibodies, including leptotene, zygotene, pachytene, and diplotene, which is consistent with the developmental stages of spermatocytes in vivo (**Figure** **5**A; Figure S1B, Supporting Information). Next, we evaluated key events of spermatocyte meiosis in TOs cultured for 21 days by comparing with in vivo, including chromosome synapsis and the occurrence of recombination/crossover. We found that the SYCP1 protein (green) signal of the central element of the synaptic complex was juxtaposed with the SYCP3 signal along the entire length of the chromosome, showing complete overlap (yellow), except in the sex chromosome region (the dotted circle in the figure indicates a part of this region), in which the colocalization of SYCP1 and SYCP3 was incomplete in the nonsynaptic fragments, indicating that spermatocytes induced in vitro can complete chromosome synapsis (Figure 5B; Figure S1C, Supporting Information). On the chromosome axis marked by SYCP3 (red), we were able to observe many discretely distributed meiosis‐specific recombinase DMC1 (green) dot‐like signals in zygotene spermatocytes, indicating that homology search and chain invasion were actively proceeding, early homologous recombination was normal, and DNA double‐strand break (DSB) repair was initiated (Figure 5C). Moreover, in pachytene spermatocytes, the γH2AX signal on the autosome axis marked by SYCP3 had disappeared and was enriched in the sex chromatin body region (marked by the dotted circle in the figure), indicating that most of the DSBs on the autosomes of pachytene spermatocytes in the TOs had been repaired (Figure 5D). However, during the pachytene stage, we observed cells with incomplete DSB repair in the TOs. These abnormal cells were characterized by the persistent presence of γH2AX signals on autosomes (Figure 5D), indicating that DNA damage repair was not yet complete. Although the proportion of normal pachytene spermatocytes in vitro was less than that in the in vivo control group (66.72% ± 3.67% vs 90.58% ± 5.57%, Figure 5E), most spermatocytes in the TOs successfully completed DSB repair and maintained normal meiotic progression. Proper meiotic DSB repair generates one to two crossovers per homologous pair, which can be marked by MutL homolog 1 protein (MLH1) foci during the pachytene stage. These crossovers are critical for maintaining bivalent integrity until the first meiotic division. Indeed, we observed MLH1 foci on chromosomes in pachytene spermatocytes, which exhibited a localization pattern similar to that observed in vivo (Figure 5F; Figure S1C, Supporting Information).

### 2.6 Air‒liquid Interface Culture Facilitates Germ Cell Maintenance in TOs

Although we referred to the conditions used for mouse OC in vitro, to further improve the maintenance of germ cells in TO, we next evaluated several key factors that may affect the maintenance of germ cells, including ECM embedding, the number and proportion of starting germ cells, and the air‒liquid interface culture method. We evaluated the maintenance of germ cells in the TOs by analyzing the ratio of TRA98^+^ cells to SOX9^+^ cells and the differentiation efficiency of germ cells in the TOs by measuring the ratio of SYCP3^+^/DDX4^+^ and H1t^+^/TRA98^+^ cells (the testis‐specific histone H1t is a marker of spermatocytes after mid‐pachytene).

First, we compared these cell aggregates with and without ECM using the protocol of Yokonishi et al^[^ ^32^ ^]^ (Figure S6A,B, Supporting Information). We found that once the cell aggregates were encapsulated by ECM, the maintenance and differentiation ratios of germ cells were significantly increased (Figure S6C, Supporting Information, bright fields; Figure S6D–F, Supporting Information, immunofluorescence (IF) fields; Figure S6G–I, Supporting Information, Statistical analysis). Quantitative immunofluorescence analysis showed that compared with no encapsulation, Matrigel encapsulation not only significantly increased the proportion of germ cells (the TRA98^+^/SOX9^+^ ratio increased from 49.74% ± 27.95% to 111.80% ± 16.47%) but also increased the proportion of meiotic positive cells (the H1t^+^ germ cell ratio increased from 2.16% ± 2.35% to 12.89% ± 4.90%) (Figure S6G,I, Supporting Information), suggesting that the enhanced physical support provided by the ECM contributes to the maintenance and differentiation of germ cells in the TOs. Notably, regardless of whether the cells were embedded in ECM, the probability of obtaining haploid spermatids after 21 days of culture was very low when the initial cell number was large (more than 200000 cells per spheroid) (Figure S6J, Supporting Information). Second, given that an oversized TO may lead to insufficient nutrient supply and hypoxia for cells in the central region, we optimized the TOs by comparing different starting cell numbers (Figure S7A, Supporting Information). Among the three different starting cell numbers tested (1000, 4000, and 20000 cells per spheroid), ≈4000 cells per TO had better germ cell maintenance and differentiation efficiency (Figure S7B–D, Supporting Information, IF fields; Figure S7E–G, Supporting Information; Statistical analysis). Quantitative immunofluorescence analysis of TOs on day 14 showed that compared with the other groups, the ratio of TRA98^+^/SOX9^+^ cells in the TOs of the 4000‐cell/sphere group reached 114.3% (90.09%‐125.6%), whereas the proportion of SYCP3^+^ spermatocytes was 83.58% (73.29%‐90.10%), and the proportion of H1t^+^ spermatocytes was 14.02% (8.84%‐19.90%) (Figure S7E–G, Supporting Information). Third, because the number of germ cells at the time of initial organoid reconstitution is based on the physiological proportion of testicular spermatogonia at day 6 in vivo, we next explored the effects of increasing the proportion of spermatogonia in organoids on subsequent differentiation and maintenance (Figure S8, Supporting Information). We sorted undifferentiated spermatogonia from mouse pup testes using an anti‐CD90.2 antibody^[^ ^33^ ^]^ (also known as Thy‐1) and compared testicular single‐cell suspensions with different spermatogonial ratios (Thy‐1^+^ cells/Thy‐1^−^ cells = 1:10, 1:30, and 1:90) to reconstitute TOs. After 21 days of culture, TOs produced with the three ratios could form tubular structures (Figure S8A, Supporting Information), but the ratio of germ cells to Sertoli cells in the 1:30 group was significantly greater than that in the 1:10 group (Figure S8B,D, Supporting Information). In particular, the proportion of H1t and TRA98 double‐positive cells in the 1:30 group was approximately 15.67% (10.00%‐22.82%), which was significantly greater than that in the 1:10 group (7.77% (2.03%‐14.12%)) and the 1:90 group (7.35% (2.54%‐16.50%)) (Figure S8C,E, Supporting Information).

**Figure 6:**
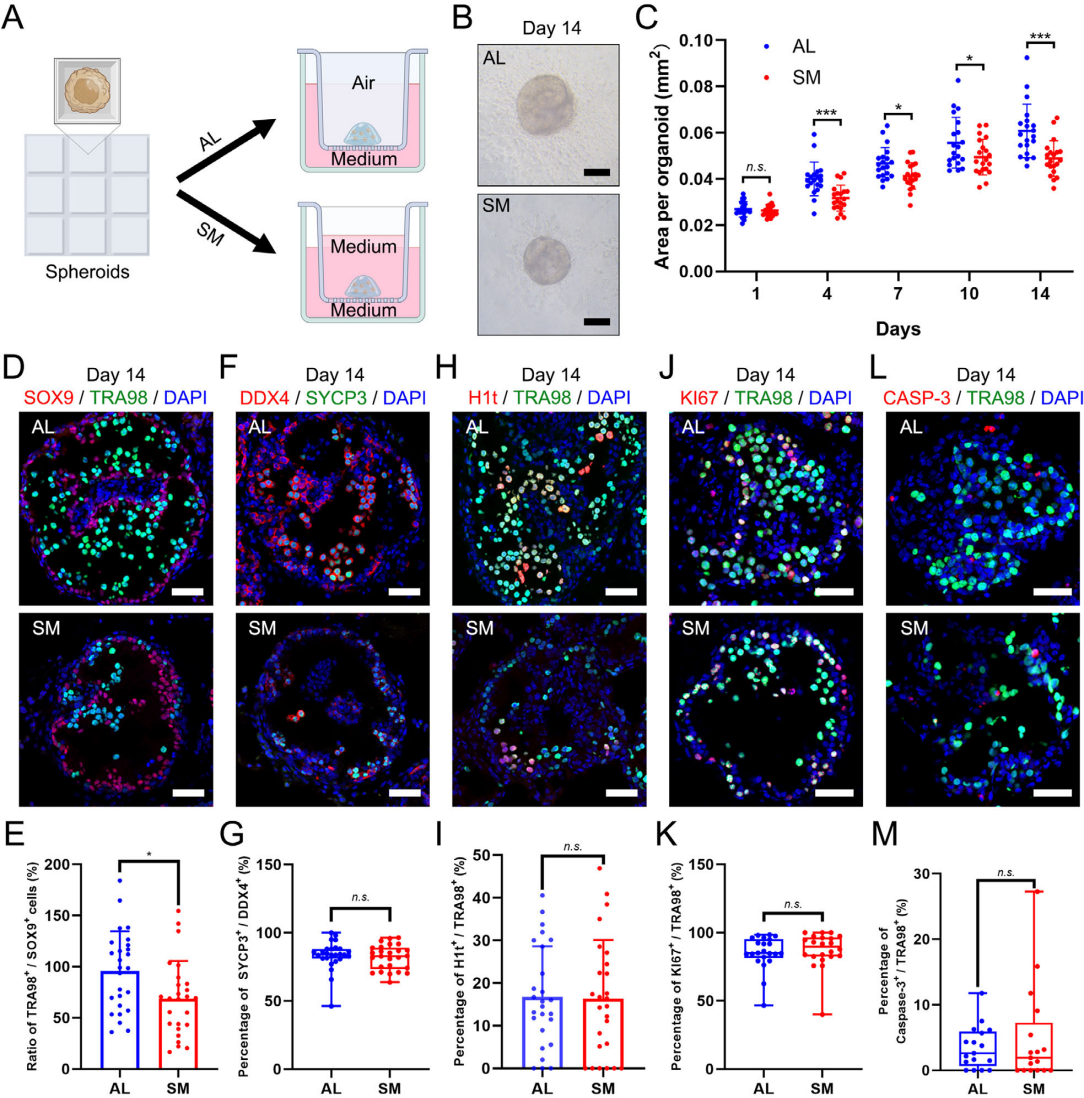
Comparison of testicular organoid (TO) development under air‒liquid (AL) and submerged (SM) culture conditions. A) Schematic illustration of the air‒liquid interface (AL) and submerged (SM) culture methods for TOs. B) Representative bright field images of TOs on day 14 of culture, showing the morphology under AL and SM conditions. Scale bar, 100 µm. C) Quantitative analysis of the organoid area (mm^2^) over a 14‐day culture period. The organoid area was measured for both the AL (blue) and SM (red) groups at specified time points. The data are presented as the means ± SDs, with individual data points shown (n = 20 organoids per group from three independent experiments). Statistical significance was determined by unpaired Student's *t*‐test. *n.s*.: not significant, * indicates *P* < 0.05, *** indicates *P* < 0.001. D–M) Immunofluorescence (IF) analysis and quantification of key cell populations and cellular processes within testicular organoids on day 14, comparing AL and SM culture conditions. D) Representative IF images showing the colocalization of Sertoli cells (SOX9, red) and germ cells (TRA98, green) within the organoids. E) Quantification of the ratio of TRA98^+^ germ cells to SOX9^+^ Sertoli cells, n = 26. F) Representative IF images demonstrating the presence of meiotic germ cells, which are marked by DDX4 (red) and SYCP3 (green). G) Quantification of the percentage of SYCP3^+^ cells among DDX4^+^ germ cells, n = 26. H) Representative IF images illustrating the presence of mid‒late pachytene spermatocytes stained with H1t (red) and TRA98 (green). I) Quantification of the percentage of H1t^+^ cells among TRA98^+^ germ cells, n = 26. J) Representative IF images assessing cell proliferation using KI67 (red) and TRA98 (green) staining. K) Quantification of the percentage of KI67^+^ cells among TRA98^+^ germ cells, n = 22. L) Representative IF images showing germ cell apoptosis induced by cleaved caspase‐3 (CASP‐3, red) and TRA98 (green). M) Quantification of the percentage of CASP‐3^+^ apoptotic cells among TRA98^+^ germ cells, n = 17. DAPI (blue) was used to stain the nuclei in all IF images. Scale bar, 50 µm. The data are presented as the means ± SDs (E and I) or median with interquartile range (G, K and M). Each dot represents an individual organoid. Statistical significance was determined by unpaired Student's *t*‐test (E and I). For the data sets in which at least one group did not follow a normal distribution (G, K and M), Mann‐Whitney U test was used. *n.s*.: not significant, * indicates *P* < 0.05.

Although an optimal system for 3D culture of testicular tubules in vitro is currently lacking, one study suggested that, compared with submerged culture (SM), air‒liquid interface (AL) culture is more conducive to the formation of testicular tubules in vitro.^[^ ^34^ ^]^ Therefore, we next compared the effects of the two culture methods on the maintenance and differentiation of germ cells in TOs (**Figure** **6**A). After 14 days of in vitro culture of TOs reconstructed from single cells from 6‐day‐old mouse testes, both culture conditions resulted in cell reorganization and tubular structure formation (Figure 6B), and the TO morphology of the SM group seemed more compact than that of the AL group. Next, quantitative analysis of the TO area of the two groups revealed that at each time point during the two‐week culture period, the TO area of the SM group was significantly smaller than that of the AL group (Figure 6C).

Subsequently, we found that on day 14 of culture in AL and SM, immunofluorescence co‐staining for SOX9 and TRA98 (Figure 6D) revealed that the germ cell maintenance rate in the AL group was significantly better than that in the SM group (95.75% ± 38.89% vs 68.80% ± 36.75%, respectively; Figure 6E). Although SYCP3‐ and H1t‐positive germ cells appeared in both groups (Figure 6F,H, Supporting Information), the proportions of SYCP3‐ and H1t‐positive germ cells did not differ significantly between the two groups (SYCP3‐positive rates were 84.02% (81.14%‐88.14%) and 82.96% (73.43%‐89.05%) in the AL and SM groups, and H1t‐positive rates were 16.79% ± 11.84% and 16.36% ± 13.76%, respectively; Figure 6G,I, Supporting Information). We continued to explore the effects of the two different culture conditions on germ cell proliferation and apoptosis. We performed immunofluorescence analysis of the cell proliferation marker KI67 and the apoptosis marker Caspase‐3 (CASP‐3) (Figure 6J,L, Supporting Information). The quantitative data revealed that there was no significant difference in the proliferation and apoptosis of germ cells between the AL and SM groups (Figure 6K,M, Supporting Information). Collectively, our findings suggest that the appropriate protocol for the maintenance and differentiation of mouse testicular germ cells in vitro via organoid culture involves the preparation of an initial cell number of 4000/spheroid, the use of a ratio of spermatogonia to total testicular cells of ≈1:30 (close to physiological conditions), and culture at the air‒liquid interface after ECM embedding.

### 2.7 Derivation of Haploid Spermatids in Chimeric Mouse TOs

**Figure 7:**
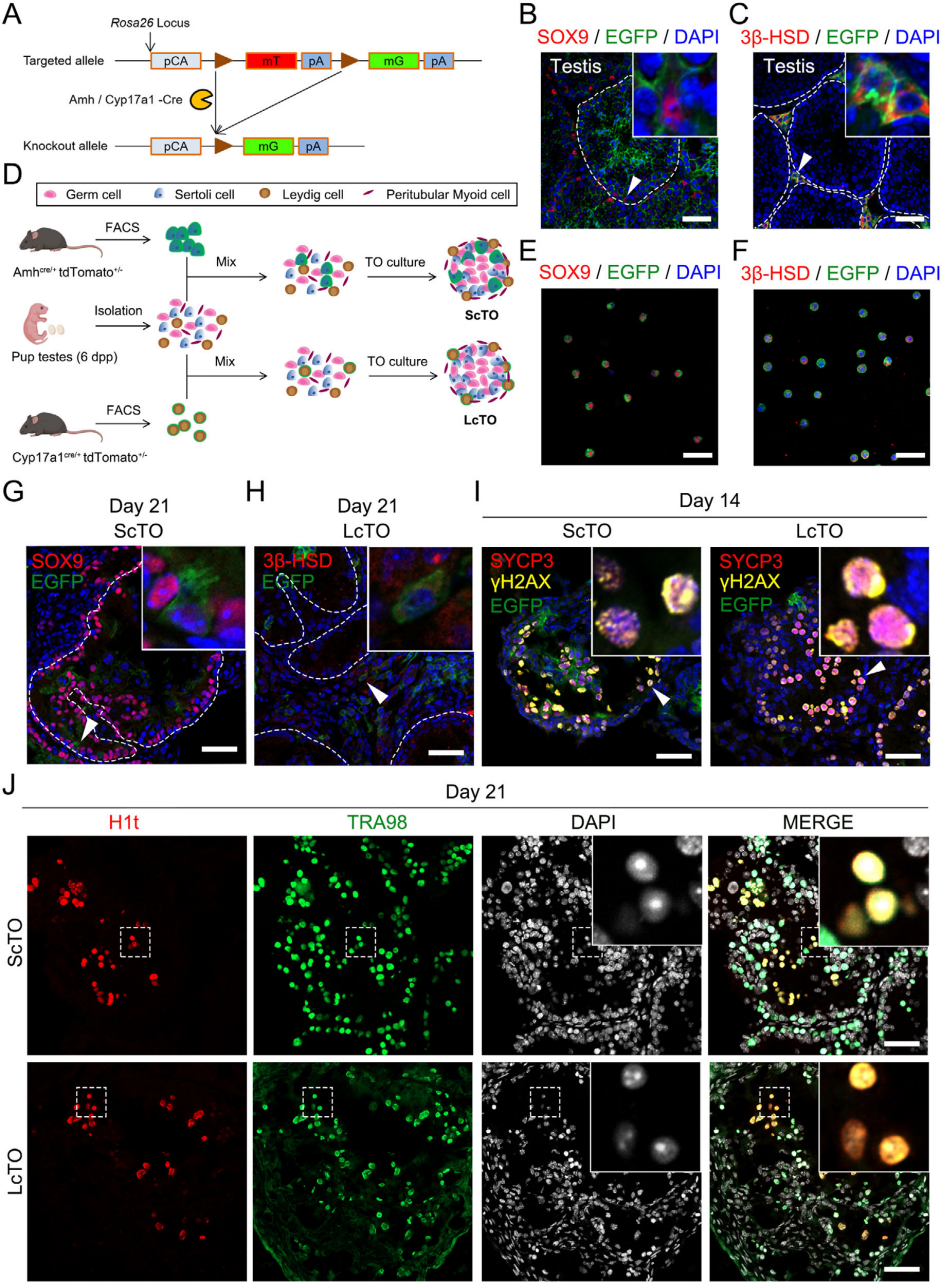
Generation and characterization of chimeric TOs. A) Schematic representation of the gene targeting strategy used to generate a conditional EGFP reporter mouse model. The targeted allele at the Rosa26 locus contains a promoter (pCA), a floxed STOP cassette (mT, encoding red fluorescent protein), and a reporter gene (mG, encoding green fluorescent protein). In the presence of Cre recombinase (Amh‐Cre for Sertoli cells and Cyp17a1‐Cre for Leydig cells) activity, the STOP cassette is excised, resulting in a knockout allele that allows for EGFP expression specifically in cells expressing Cre recombinase. B) Immunofluorescence of mouse (Amh^Cre/+^; tdTomato^+/−^) testis sections showing EGFP autofluorescence (green) in Sertoli cells labeled with SOX9 (red). DAPI (blue) was used to stain the nuclei. Scale bar, 50 µm. C) Immunofluorescence of mouse (Cyp17a1^Cre/+^; tdTomato^+/−^) testis sections showing EGFP autofluorescence (green) in Leydig cells labeled with 3ß‐HSD (red). DAPI (blue) was used to stain the nuclei. Scale bar, 50 µm. D) Schematic diagram of the protocol for generating chimeric TOs by enriching EGFP‐expressing Sertoli cells (ScTO) or Leydig cells (LcTO) by fluorescence‐activated cell sorting (FACS) and then mixing them with unsorted testicular cells. E) Representative images of FACS‐sorted Sertoli cells from mouse (Amh^Cre/+^; tdTomato^+/−^) testes. EGFP (green) and SOX9 (red) indicate the purity of the sorted Sertoli cell population, and DAPI (blue) was used to stain DNA. Scale bar, 50 µm. F) Representative images of FACS‐sorted Leydig cells from mouse (Cyp17a1^Cre/+^; tdTomato^+/−^) testes. EGFP (green) and 3β‐HSD (red) indicate the purity of the sorted Leydig cell population, and DAPI (blue) was used to stain DNA. Scale bar, 50 µm. G) Immunofluorescence staining of ScTOs after 21 days of culture revealed the presence of fluorescent Sertoli cells (EGFP‐positive, green) in chimeric TOs. Scale bar, 50 µm. H) Immunofluorescence staining of LcTOs after 21 days of culture revealed the presence of fluorescent Leydig cells (EGFP‐positive, green) in chimeric TOs. Scale bar, 50 µm. I) Immunofluorescence staining of ScTOs and LcTOs after 14 days of culture revealed the presence of meiotic spermatocytes. SYCP3 (red) and γH2AX (yellow) labeled spermatocytes. DAPI (blue) was used to stain the nuclei. Scale bar, 50 µm. J) Immunofluorescence staining of ScTOs and LcTOs after 21 days of culture revealed the presence of mid‒late pachytene spermatocytes and postmeiotic germ cells. H1t (red) marks mid‒late pachytene spermatocytes and postmeiotic germ cells, and TRA98 (green) marks the germ cells. DAPI (white) was used to stain the nuclei. Scale bar, 50 µm.

One potential application of TOs is to control and modify the properties of different cell types prior to self‐assembly to explore the feasibility of IVS, but this requires integrating cells from multiple different donors into genetically chimeric TOs (or chimeras). To test this application, we first mated Gt(ROSA)26Sor^tm^ ^4(ACTB‐tdTomato,‐EGFP)Luo^/J transgenic mice (also called ROSA^mT/mG^, Strain#:007576, Jackson Laboratory) with Amh‐Cre (Sertoli cell‐specific Cre recombinase) transgenic mice or Cyp17a1‐Cre (Leydig cell‐specific Cre recombinase) transgenic mice to obtain mice in which testicular Sertoli cells specifically expressed green fluorescent protein (Amh^Cre/+^; tdTomato^+/−^) or testicular Leydig cells specifically expressed green fluorescent protein (Cyp17a1^Cre/+^; tdTomato^+/−^) (**Figure** **7**A–C). Then, green fluorescent Sertoli cells or Leydig cells were obtained from 12‐day‐old transgenic mice by fluorescence‐activated cell sorting (FACS) and mixed into the testicular single‐cell suspensions of 6‐day‐old nontransgenic ICR mice at a ratio of 1:10 for TO construction and culture (Figure 7D–F).

Next, we observed chimeric mouse TOs formed by Sertoli cells (ScTO) or Leydig cells (LcTO) derived from different genetic backgrounds (Figure 7D). After 21 days of culture, we observed that green fluorescent and SOX9‐positive Sertoli cells in ScTOs are located in the periphery of the tubular structure (Figure 7G). Similarly, in LcTOs, green fluorescent and 3β‐HSD‐positive Leydig cells were observed in the interstitium between the tubular structure (Figure 7H). Next, we performed SYCP3 and γH2AX immunofluorescence staining on ScTOs and LcTOs cultured for 14 days, and spermatocytes that entered meiosis were found in both types of organoids (Figure 7I). In addition, we performed H1t and TRA98 immunofluorescence staining on ScTOs and LcTOs cultured for 21 days, mid‒late pachytene spermatocytes and haploid spermatids (the nuclei localized with DAPI are clearly haploid round spermatids) that developed from spermatogonia from 6‐day‐old mice were found to be double‐positive for H1t and TRA98 in both organoids (Figure 7J). Together, these results indicate that chimeric mouse TOs derived from the mixing and reconstitution of single‐cell suspensions of germ and somatic cells from different mouse backgrounds can also support germline development into spermatid‐like haploid cells.

## 3 Discussion

In this study, our TOs reconstituted from single‐cell suspensions of 6‐day‐old neonatal mouse testes were able to form ZO‐1‐ and CLDN11‐positive tubular structures and tight junctions. Importantly, germ cells (undifferentiated spermatogonia) in the TOs were able to maintain their existence and continue to differentiate. Moreover, the temporal and spatial order of their in vitro development was similar to that of their in vivo development, and most of the key steps of IVS were completed. Single‐cell sequencing analysis revealed that possible haploid spermatids accounted for at least 2.43% of all germ cells, and this proportion may have been higher in some batches of TOs, as determined by immunofluorescence staining of the acrosome‐specific marker PNA. Notably, although the germ cells in TOs that enter meiosis can develop into spermatocytes in the leptotene, zygotene, pachytene and diplotene stages, complete the core events in meiosis (chromosome synapsis and recombination), and develop into haploid spermatids, in the current culture system, the development rate of germ cells is still much lower than that of somatic cells, especially those with a large number. Overcoming the problem of germ cells being surpassed by somatic cells is still a key step in improving the efficiency of this process. Recent studies in TOs have reported on the key stages of spermatogenesis in vitro, such as meiosis,^[^ ^21^, ^35^ ^]^ whereas a unique advance of our current work is the comparable description of the spatiotemporal dynamics of spermatogenesis in testicular organoids.

Organoids with complex structures and biomimetic tissue functions have always been considered extremely challenging and difficult to control because they need to meet the three defining benchmarks of organoids: self‐assembly, complex internal morphology, and tissue function.^[^ ^36^ ^]^ Similarly, the construction of biomimetic testes with spermatogenic function in vitro remains a challenge, and previous TO‐based IVS studies using testicular cell suspensions have attempted to obtain postmeiotic germ cells but have rarely focused on improving testicular structural reconstruction.^[^ ^37^ ^]^ The success of testicular OC highlights that maintenance of spatial organization is a key factor in IVS.^[^ ^38^ ^]^ However, although a variety of culture methods have been explored to promote testicular structural reconstruction, few methods can reproduce all the main characteristics of the testis,^[^ ^37^, ^39^ ^]^ including tubular structure development; the cell arrangement; the maintenance and differentiation of germ cells; and the establishment and maintenance of the blood‒testis barrier. In recent years, a variety of different culture methods, including the use of scaffold‐free cultures or scaffold‐based cultures, have been attempted in humans, mice, pigs, and rats. However, compared with the research progress on organoids in other tissues, the exploration of TOs has been limited.^[^ ^37^, ^40^ ^]^ The scaffold‐free testicular organoid culture system developed by Yokonishi et al. represents a promising model for IVS.^[^ ^32^ ^]^ In their study, single cells from neonatal mouse testes reorganized into tubular structures in vitro without exogenous ECM support, and although germ cells showed differentiation capacity, their development was reportedly blocked at the pachytene spermatocyte stage.^[^ ^32^ ^]^ In contrast, the TOs constructed in this study seem to have more advantages in terms of germ cell development.

Germ cell development continues throughout the lifespan. Mouse primordial germ cells (PGCs) are induced from the proximal ectoderm at embryonic day 6.25 (E6.25) and undergo sex determination in the mouse gonad in response to somatic cell signals generated at E12.5. Next, XY‐type PGCs can transform into prospermatogonia and gradually re‐enter the cell cycle between 1.5 and 3.5 days after birth, ultimately transforming into a pool of SSCs that support spermatogenesis throughout the lifespan.^[^ ^41^, ^42^ ^]^ It takes ≈19 to 21 days for prospermatogonia and SSCs in the mouse testis (first and subsequent wave of spermatogenesis) to differentiate into haploid spermatids and ≈35 days to finally develop into sperm, whereas spermatogenesis in humans after puberty takes 72–73 days.^[^ ^43^ ^]^ Interestingly, this in vivo process appears to be significantly accelerated in vitro in 2D culture systems. A previous study by Zhou et al. revealed that mouse spermatid‐like cells (SLCs) can be obtained through the stepwise differentiation of ESCs, and the entire process takes ≈22 days;^[^ ^9^ ^]^ another study by Hayashi et al. revealed that epiblast‐like cells (EpiLCs) obtained via the induction of E3.5 blastocyst‐derived ESCs for 3 days or EpiLCs obtained via the induction of iPSCs for 2 days require up to 6 days to differentiate into primordial germ cell‐like cells (PGCLCs) and then can fully recapitulate spermatogenesis similar to that of SSCs through in vivo transplantation.^[^ ^44^ ^]^ On the other hand, IVS attempts using postnatal testes or germ cells have shown inconsistent times for the development of SSCs or undifferentiated spermatogonia into haploid cells in various culture systems (e.g., agarose embedding, organ‐on‐a‐chip, or organoid approaches), ranging from 3 to 7 weeks.^[^ ^21^, ^45^, ^46^, ^47^, ^48^, ^49^ ^]^ In contrast, the organoids in this study required ≈1 week to reconstruct the testicular single cells embedded in the ECM, and the time for each stage of germ cell development in vitro was then comparable to that in vivo, indicating that the intrinsic developmental program of male germ cells can proceed normally even in the absence of microenvironmental conditions with precise regulation of in vivo signals. Nevertheless, it should be noted that the organoids we constructed support the development of haploid spermatids through only the same 4/5 steps as spermiogenesis in vivo, and how to achieve complete all steps of spermiogenesis in organoids remains a difficult problem.

In our pursuit of haploid spermatids generation, we found that Matrigel embedding and air‐liquid interface cultures are crucial for advancing IVS. These two factors synergistically promote meiotic progression. Sakib et al. previously proposed that testicular cells can spontaneously aggregate into spheres after being seeded on microwell plates, but their pattern is flipped; that is, germ cells and supporting cells are located around the basement membrane, whereas interstitial cells and peritubular muscle cells are located in the tubular structure.^[^ ^22^ ^]^ We used the same microsphere formation method but found that the constructed TOs presented an arrangement highly similar to that in vivo ≈1 week after single‐cell reconstitution. We speculate that this significant difference is due to the ECM embedding after sphere formation, and the interaction between testicular cells and the ECM greatly improves their reconstruction efficiency. ECM is widely used in the construction of various tissues and organs because of its good biocompatibility.^[^ ^50^ ^]^ In addition to providing mechanical support for cells, it can also activate a variety of biochemical signals and promote cell reorganization in vitro,^[^ ^51^ ^]^ thereby yielding a 3D structure closer to that in vivo, which is also a prerequisite for reproducing the corresponding tissue function in vitro. However, there is still controversy as to whether the formation of TOs requires a supporting scaffold or ECM components.^[^ ^22^, ^32^, ^39^, ^52^ ^]^ Our results indicate that ECM embedding technology significantly increased the efficiency of germ cell meiosis.

The air‒liquid interface culture method, considered the gold standard for organ culture, originated in the early 20th century and was finalized by Trowell in the late 1950s.^[^ ^53^ ^]^ Despite minor subsequent adjustments, the technique has remained substantially unchanged. The core principle of this culture method is to enhance tissue oxygen accessibility, thereby alleviating tissue degeneration and necrosis. In this study, using a testicular organoid model, we revealed the advantages of air‒liquid interface culture over conventional submerged culture in germ cell maintenance and differentiation. Under submerged culture conditions, a significantly elevated number of germ cells underwent apoptosis, suggesting their susceptibility to hypoxia, consistent with previous findings.^[^ ^54^, ^55^ ^]^ In addition, owing to the lack of a circulatory system, larger organoids inevitably suffer from necrosis caused by hypoxia in the center of the tissue. Previous studies have shown that the integrity of the proper exchange of oxygen and nutrients is optimal when the diameter of the testis fragments in organ culture is 300–400 µm.^[^ ^56^ ^]^ Therefore, we set a series of cell concentration gradients to explore the effect of organoid size on germ cell development. Bright field imaging indicated that reducing organoid size alleviated central degeneration in TOs. However, it is noteworthy that germ cell development in the group with an initial cell density of 1000 cells/spheroid was markedly less robust compared to the group with 4000 cells/spheroid. We speculate that insufficient cell numbers may lead to inadequate endogenous ECM secretion, resulting in a structurally compromised basement membrane that fails to sustain the spermatogenic microenvironment. Regardless, in our study, we speculate that the primary role of the ECM in our system may be to provide a supportive scaffold that promotes tissue maintenance which is essential for cell survival and development.^[^ ^57^ ^]^

To explore the nutritional system required for IVS in organoids, we used MEMα + 10% KSR or MEMα + AlbuMAX (40 mg mL^−1^) according to the testicular OC method reported by Sato et al.^[^ ^14^ ^]^ (Tables S1 and S2, Supporting Information), and both culture media were able to achieve OC‐derived IVS and produce elongated spermatids at approximately day 28 of culture. However, when we applied these two culture media systems for the generation of TOs, neither medium alone could well support the undifferentiated spermatogonia derived from 6‐day‐old testes in undergoing meiosis and developing into haploid spermatids (Figure S2A, Supporting Information). Indeed, previous studies have shown that KSR facilitates the structural reorganization of testicular cells,^[^ ^37^, ^58^ ^]^ and it has thus been used as a primary component in culture medium for TOs. To further improve the composition of the culture medium, we explored various additives to enhance IVS in TO experiments, referring to other reports.^[^ ^9^, ^59^, ^60^ ^]^ We found that the efficiency of meiosis in germ cells within the TOs was significantly enhanced by the addition of a combination of hormones (Follicle‐stimulating hormone and testosterone) and antioxidants (α‐tocopherol, ascorbic acid, and glutathione) (Figure S2A, Supporting Information). Nevertheless, it should be pointed out that the organoids we constructed only support the development of haploid spermtids to the same step‐4/5 as spermiogenesis in vivo. We postulate that this phenomenon could be attributed to the fact that the reconstructed architecture of TOs does not yet fully recapitulate the arrangement of spermatogenic cells in the seminiferous tubules of the testis in vivo. An alternative explanation is the incomplete maturation of the enclosed testicular somatic cells, potentially caused by a suboptimal nutritional support. Interestingly, we found that the addition of AlbuMAX or lysophospholipids to 10% KSR resulted in poor germ cell maintenance. This may be because germ cells are sensitive to direct exposure to these nutrients. Instead, their development should be indirectly promoted through the nourishment of testicular somatic cells, a process that can only occur after the testicular structure has been reorganized. Therefore, to enhance the yield of haploid spermatids and achieve complete IVS, it is essential to explore a culture system utilizing a sequential media formulations. Finally, we acknowledge that functional validation of the in vitro‐derived haploid spermatids, one of the gold standards for verifying gametogenesis,^[^ ^61^ ^]^ has yet to be performed. Nevertheless, we not only detected haploid spermatids through histological and immunofluorescence analyses but also demonstrated the presence of round spermatid‐specific molecular markers within the germ cell population of TOs using single‐cell RNA sequencing. More importantly, chromosomal spreads demonstrated that key meiotic events could occur normally in vitro. Future efforts should concentrate on optimizing culture medium to increase haploid spermatids yield and promote spermiogenesis. Furthermore, we have not yet determined the initial cell types and ratios used for organoid culture in this study. Knowing the initial ratios of each cell type may help us better understand this model and its in vitro development.

In summary, this study established a TO culture system that can produce haploid spermatids. Combined with the rapid development of stem cell manipulation and gene modification technologies, this system will become a tool for studying gene function during spermatogenesis and enabling patients with genetic diseases to obtain healthy sperm.^[^ ^62^ ^]^ This study also provides a potential research platform for drug screening, reproductive toxicity studies and other studies.

## 4 Experimental Section

### Animals

All the mice were housed under specific pathogen‐free conditions and maintained on a 12‐h light/dark cycle with food and water ad libitum. All the experimental procedures were carried out in accordance with the guidelines of the Institutional Animal Care and Use Committee of Nanjing Medical University (Approval number: IACUC‐1911006). All the mouse experiments were conducted with 6‐day postpartum (dpp) ICR mice, except for the generation of chimeric TOs.

The chimeric TOs experiments were performed additionally using Gt(ROSA)26Sor^tm^ ^4(ACTB‐tdTomato,‐EGFP)LUO^/J transgenic mice (007576, The Jackson Laboratory), a double‐fluorescent Cre reporter mouse that expresses membrane‐targeted tdTomato (mT) prior to Cre‐mediated excision and membrane‐targeted green fluorescent protein (mG) after excision.^[^ ^63^ ^]^ To achieve cell‐specific expression of mG, Gt(ROSA)26Sor^tm^ ^4(ACTB‐tdTomato,‐EGFP)LUO^/J transgenic mice were crossed with Amh‐Cre and Cyp17a1‐Cre (C001049, Cyagen) transgenic mice. The Amh‐Cre mice were shared by Dr Guo Xuejiang at State Key Laboratory of Reproductive Medicine and Offspring Health, Nanjing Medical University.

### Organ Culture

Immature testes were obtained from ICR mice at 5–6 dpp. The testes were subsequently decapsulated and rinsed in ice‐cold HBSS (Gibco, 14175‐095). The testes were divided into fragments ≈1–3 mm in diameter and transferred onto 1.5% agarose gel blocks that had been preequilibrated in culture medium for 24 h. One agarose gel block was placed into each well of a 12‐well plate and loaded with 1–3 tissue fragments. The culture medium was MEMα (Gibco, 12561‐049) supplemented with 10% KSR (Gibco, 10828‐028) or 40 mg mL^−1^ AlbuMax I (Gibco, 11020021). Approximately 0.5 mL of culture medium was added to each well of a 12‐well plate and incubated at 34 °C. The culture medium was replaced once a week (see Tables S1 and S2, Supporting Information for specific culture media and components).

### Isolation of Testicular Cells

Testes from 5–6 dpp ICR mice were collected and then subjected to a two‐step enzymatic hydrolysis according to previous methods and procedures.^[^ ^33^, ^64^ ^]^ Briefly, testis tissues were placed in MEMα (Gibco, 12561‐049) containing 1 mg mL^−1^ collagenase I (Sigma, C5138) and 5 µg mL^−1^ deoxyribonuclease I (Sigma, DN25), gently triturated 50 times and incubated for 8 min at 37 °C. Then, hyaluronidase (Sigma, H3506) was added to a final concentration of 1 mg mL^−1^, and the cell suspensions were triturated and incubated for another 10 min at 37 °C. The reaction was stopped by adding fetal bovine serum (Gibco, 10099–141) to a final concentration of 10%. The cells were filtered through a membrane with a pore size of 40 µm (JET Biofil, CSS013040). The cells were subsequently centrifuged at 600 × g for 5 min to pellet the cells and resuspended in SSC culture medium^[^ ^33^ ^]^ (MEMα (Gibco, 12561‐049), 1% penicillin/streptomycin (Gibco, 15140‐122), 0.2% bovine serum albumin (Sigma, A3803), 10 µg mL^−1^ Transferrin (Sigma, T1283), 3 × 10^−8^ M sodium selenite (Sigma, 481815), 2 mM GlutaMAX (Gibco, 35050–061), 50 µM 2‐mercaptoethanol (Sigma, M7522), 5 µg mL^−1^ insulin (Sigma, I5500), 10 mM HEPES (Sigma, H0887), and 60 µM putrescine (Sigma, P5780)) supplemented with recombinant human glial cell line‐derived neurotrophic factor (10 ng mL^−1^, R&D Systems), rat recombinant GFRα1/Fc (150 ng mL^−1^, R&D Systems) and chimeric human recombinant bFGF (1 ng mL^−1^, Corning).

### Generation of TOs

The microwells (Shenzhen Xirui Biotech Inc., CAwell600) were placed into a 24‐well plate, prepared per the manufacturer's instructions, and washed three times with 0.5 mL of PBS before cell seeding. A total of 6.0 × 10^6^ (20000 cells/organoid), 1.2 × 10^6^ (4000 cells/organoid), or 3.0 × 10^5^ (1000 cells/organoid) testicular cells in 1 mL of SSC culture medium supplemented with 2% BME (Bio‐Techne Co., 3533) were placed into each well and kept at room temperature after cell seeding for 10 min. The plate was then centrifuged at 600 × g for 5 min at 4 °C. The cells were cultured in microwells at 37 °C and 5% CO_2_ for 24 h to form spheroids. The next day, the spheroids were collected and centrifuged at 200 × g for 2 min. After the supernatant was removed, the spheroids were resuspended in BME diluted 3:1 in ice‐cold MEMα and 1% Pen–Strep, after which they were added dropwise (20 µL per drop) to the apical chamber of a transwell (JET BIOFIL, TCS016012). Each 20 µL drop contained ≈20–30 spheroids. The transwells were incubated in a 37 °C incubator to allow the BME to solidify. After the BME solidified for half an hour, ≈0.5 mL of culture medium was added to the outer chamber of each well of a 12‐well culture plate equipped with an insert and cultured at 34 °C. On day 14, organoids were recovered from the ECM using commercial cell recovery solution (Corning, 354253) according to the manufacturer's instructions. The organoid culture medium consisted of MEMα, 1% (vol/vol) Pen–Strep, 10% KSR (Gibco, 10828‐028), 500 mM L‐ascorbic acid 2‐glucoside (TCI, G0394), 500 mM DL‐alpha‐tocopherol acetate (Merck, T3376), 500 mM reduced L‐glutathione (Merck, G6013), 1 ng mL^−1^ follicle‐stimulating hormone from the human pituitary (Merck, F4021), and 1 µM testosterone (Merck, T037). The culture medium was replaced every 4 days (see Table S3, Supporting Information for specific culture media and components).

### Histological and Immunofluorescence Analyses

The organoids and cultured testicular tissues were placed in Hartman's fixative and fixed for 2 h at room temperature for histological analysis. The fixed samples were embedded in paraffin wax and sectioned at 5 µm. These sections underwent deparaffinization and rehydration treatment and were finally stained with hematoxylin and eosin (H&E). At the end of the culture period, the organoids and cultured testicular tissues were fixed in 4% paraformaldehyde in PBS for 30 min at room temperature, embedded in O.C.T. (SAKURA, 4583) and cut into 7 µm thick sections. The slides were washed with PBS, blocked with blocking solution containing 10% normal donkey serum and 3% BSA in PBS for 40 min at room temperature and incubated with primary antibodies against GATA4 (1:200, Santa Cruz Biotechnology, sc‐1237), 3β‐HSD (1:200, Proteintech, 15516‐1‐AP), α‐SMA (1:200, Proteintech, 14395‐1‐AP), ZO‐1 (1:200, Proteintech, 21773‐1‐AP), Claudin‐11 (1:400; Affinity, AF5364), SOX9 (1:1000, Millipore, AB5535), TRA98 (1:2000, Abcam, ab82527), DDX4 (1:500, Abcam, ab13840), PLZF (1:400, R&D Systems, AF2944), STRA8 (1:200, Abcam, ab308124), c‐KIT (1:200, R&D Systems, AF1356), SYCP3 (1:400, Abcam, ab97672), γH2AX (1:500, Abcam, ab11174), H1t (1:500, ABclonal, A18597), CREM (1:200, Proteintech, 12131‐1‐AP), Caspase‐3 (1:200, Proteintech, 25128‐1‐AP), KI67 (1:1000, Abcam, ab15580), AR (1:200, Abcam, ab133273). After overnight incubation at 4 °C, the slides were washed three times in PBS, followed by incubation with secondary antibodies (Jackson ImmunoResearch) or/and peanut agglutinin (PNA) (1:1000, Vectorlabs, RL‐1072) and DAPI (1:500, Sigma, D9542) for 2 h at room temperature, followed by three washes with PBS. For the background control, normal rabbit IgG, normal goat IgG or normal mouse IgG was used instead of the primary antibody (see Table S5, Supporting Information for primary antibodies information). Images were captured with an LSM‐800 inverted confocal microscope (Zeiss). For BTB function assays, the Evans Blue method was followed.^[^ ^65^ ^]^ Briefly, organoids cultured for 1, 7, 14, or 21 days were exposed to Evans Blue (Sigma. E2129) for 30 min and then imaged immediately (seminiferous tubules from 7‐, 14‐, and 21‐day‐old mice were used as controls).

### Spermatocyte Nuclear Spreading

TOs were digested into single‐cell suspensions by two‐step enzymatic dissociation. Chromosomal spread preparations were prepared as previously described.^[^ ^66^ ^]^ The cells were fixed, permeabilized, and blocked as previously described, followed by overnight incubation at 37 °C with primary antibodies (see Table S5, Supporting Information for primary antibodies information). The primary antibodies used were anti‐SYCP3 (1:200, Abcam, ab97672), anti‐γH2AX (1:1000, Abcam, ab11174), anti‐SYCP1 (1:200, Abcam, ab15090), anti‐DMC1 (1:50, Proteintech, 13714‐1‐AP), and anti‐MLH1 (1:50, Proteintech, 11697‐1‐AP). The next day, the slides were washed with TBS‐T (0.1% Triton X‐100) three times and incubated with the corresponding secondary antibodies (Jackson ImmunoResearch) and DAPI. The images were captured with an LSM‐800 inverted confocal microscope (Zeiss).

### Magnetic‐Activated Cell Sorting (MACS)‐Mediated Germ Cell Enrichment

Thy‐1^+^ germ cells were captured from the total testicular cell suspensions using CD 90.2 microbeads (Miltenyi Biotec, 130‐121‐278) for MACS in accordance with the manufacturer's instructions. Briefly, the single‐cell suspension was centrifuged at 600 × g for 5 min and resuspended in 200 µL of PBS‐S (Dulbecco's PBS (Gibco, 14190250) supplemented with 1% FBS (Gibco, 10099‒141), 10 mM HEPES (Sigma, H0887), 1 mg mL^−1^ D(+)‐glucose (Sigma, G8270), 1 mM sodium pyruvate (Gibco, 11360070), and 1% penicillin/streptomycin (Gibco, 15140‒122)). Next, 20 µL of CD90.2 microbeads were added per 10^7^ cells at 4 °C for 20 min. The unbound beads were removed by removing the supernatant after centrifugation at 600 × g for 5 min. The cell pellet was then resuspended in 1 mL of PBS‐S and transferred into an MS column prerinsed with PBS‐S. Both the Thy‐1^+^ and Thy‐1^+^‐depleted cell fractions were collected, followed by three washing steps with 500 µL of PBS‐S. Thy‐1^+^ cells were then combined with Thy‐1^+^‐depleted cells at ratios of 1:10, 1:30, and 1:90 to the total cells. These cells were seeded in microwells to produce organoids as previously described.

### Single‐cell Preparation for FACS and scRNA‐seq

Single cells were isolated according to a previous protocol^[^ ^67^ ^]^ with slight modifications. Briefly, decapsulated mouse testes and day 28 TOs were collected and washed twice in HBSS (Gibco, 14175‐095). Testes and TOs were digested in 3 mL of MEMα containing 1 mg mL^−1^ collagenase I (Sigma, C5138) and 5 µg mL^−1^ deoxyribonuclease I (Sigma, DN25), gently triturated 50 times and incubated for 8 min at 37 °C. The dispersed tissue was then centrifuged at 600 × g for 5 min and digested with 0.25% trypsin/EDTA (Gibco, 25200‐072) and deoxyribonuclease I (Sigma, DN25) at 37 °C with gentle agitation for 4 min. The enzymatic reaction was neutralized by the addition of FBS at a final concentration of 10%. The cells were then filtered through a 40 µm filter to remove cell aggregates. Cell viability was examined by a 0.4% trypan blue exclusion assay (Gibco, 15250‐061) in a Neubauer chamber (Neubauer, Blaubrand, Germany). The viability of the cells exceeded 90%, and the aggregation rate was less than 5%. The cell suspension was washed twice with DPBS (calcium‐ and magnesium‐free PBS) and resuspended in DPBS + 0.04% BSA at a cell concentration of 1000 cells/µL.

### scRNA‐seq Library Preparation

During library preparation, individual gel beads and single cells are encapsulated within discrete oil‒water emulsion droplets to form gel beads‐in‐emulsion (GEMs). Each gel bead carries unique molecular identifiers, including a cell barcode sequence, unique molecular identifiers (UMIs), and poly‐dT primer sequences that initiate reverse transcription. In this GEM reaction system, cells subsequently undergo lysis, releasing mRNA molecules that hybridize to the poly‐dT primers. Under the catalysis of reverse transcriptase, first‐strand cDNA synthesis is initiated, generating full‐length cDNA molecules. These cDNA molecules are then amplified and processed for library construction.

### Cell Type Identification on the Basis of Single‐Cell RNA‐seq Data

Cell type identification was performed according to the expression levels of multiple canonical marker genes. Initially, cells are divided into germ cells and somatic cells on the basis of the expression of *Ddx4* (for germ cells) and *Vim* (for somatic cells). For somatic cells, Sertoli cells were characterized by high expression of *Sox9*, *Wt1*, and *Cldn11*. Leydig cells were identified by *Cyp11a1*, *Cyp17a1*, and *Insl3* expression. The macrophages were annotated using *Ptprc*, *Cd68*, and *Cd74*, and the endothelial cells (ECs) were identified by *Vwf* and *Pecam1*. For spermatogenic cells, spermatogonia were labeled with *Zbtb16*, *Stra8*, and *Esx1*; spermatocytes were labeled with *Meiob*, *Piwil1*, and *Cdk1*; and spermatids were labeled with *Tex36*, *Tnp1*, and *Prm1*. This approach allowed accurate classification of cell types on the basis of their distinct gene expression profiles.

### Processing of Single‐Cell RNA‐seq Data

The feature‒barcode matrices for each sample were generated using Cell Ranger (version 7.1.0). The doublet cells were removed from the data using the DoubletFinder package (version 2.0.3). Cells were excluded from further analysis on the basis of the following criteria: fewer than 1000 or more than 5000 detected genes, total RNA counts exceeding 20000, or a percentage of mitochondrial RNA greater than 10%. Only the remaining cells that met these quality control standards were retained for subsequent analyses. Gene expression normalization was performed using the “NormalizeData” function with the parameters “normalization.method = “LogNormalize”, scale.factor = 10000” in the Seurat package (version 4.4.0). Principal component analysis (PCA) was performed using the “RunPCA” function. Batch effect correction was conducted using the Harmony package (version 0.1.1). Then, the results generated by “RunHarmony” were further processed with “FindNeighbors” and “RunUMAP” for cell clustering. The DEGs were identified using the “FindMarkers” function in the Seurat package (version 4.4.0) with the Wilcoxon rank‐sum test. The DEGs were filtered on the basis of adjusted *P* values less than 0.01 and absolute fold changes greater than 2. Additionally, the gene expression difference was required to exceed the first quartile of the ranked average expression levels of all genes. For functional enrichment analysis, the clusterProfiler (version 4.6.2) package was used to perform Gene Ontology (GO) enrichment analysis and KEGG pathway enrichment analysis of the identified DEGs.

### Flow Cytometry

Single‐cell suspensions from 12 dpp transgenic mice (Amh^Cre/+^; tdTomato^+/−^) or (Amh^Cre/+^; tdTomato^+/−^) were obtained as previously described. GFP‐expressing Sertoli/Leydig cells were sorted using a FACSAria Fusion SORP flow cytometer (BD Biosciences) and analyzed using FlowJo software (BD Biosciences, version 8.0.3). To generate chimeric organoids, Sertoli/Leydig cells were mixed with 6 dpp ICR whole testicular cells at a ratio of 1:10 to total cells. These cells were seeded in microwells to produce organoids as previously described.

### RNA Extraction and Quantitative Real‐Time (qRT)‐Polymerase Chain Reaction (PCR)

Total RNA was extracted from TOs and testicular tissue using TRIzol Reagent (Invitrogen) following the manufacturer's protocol. Briefly, dissociated single cells from the organoids and testes were homogenized in TRIzol at room temperature for 5 min. Phase separation was achieved by adding trichloromethane, mixing thoroughly, and centrifuging at 12000 × g for 15 min at 4 °C. The upper aqueous phase containing RNA was transferred to a new tube and mixed with isopropanol to precipitate the RNA. Following centrifugation at 12000 × g for 10 min at 4 °C, the supernatant was discarded. The RNA pellet was washed with 75% ethanol, air‐dried briefly, and resuspended in 30 µL of RNase‐free DEPC‐water before being stored at −80 °C. For gene expression analysis, mRNA was first reverse‐transcribed into cDNA using the PrimeScript RT Master Mix (TAKARA). Subsequently, quantitative PCR (qPCR) was performed with ChamQ SYBR qPCR Master Mix (Vazyme Biotech) on a CFX Connect Real‐Time PCR Detection System (Bio‐Rad) following the manufacturer's instructions. Relative gene expression levels were calculated using the 2^−∆∆Ct^ method, with *Actin* serving as the normalization control. RNA extracted from primary testicular cells from neonatal mice (6 dpp) served as a control. Primer sequences were listed in Table S4 (Supporting Information).

### Statistical Analysis and Image Layout

All single‐cell sequencing experimental results were obtained from organoids collected from two independent batches. Unless otherwise specified, at least three independent replicates were performed for other experiments. All the results obtained are expressed as the means ± SDs or median with interquartile range and *P* < 0.05 was considered to indicate statistical significance, * indicates *P* < 0.05, ** indicates *P* < 0.01, and *** indicates *P* < 0.001. Unpaired t‐tests were used to analyze the differences in normally distributed data compared between 2 groups, and one‐way Analysis of Variance (ANOVA) with Tukey's multiple comparisons test was used in data with ≥3 groups. For data sets in which at least one group did not follow a normal distribution, the Mann‐Whitney U test was used. The software used for data analysis was GraphPad Prism 8 (version 8.0.2), and that used for image layout was Adobe Photoshop 2021 (version 22.0.0). Schematic diagrams were created using BioRender. com.

## Conflict of Interest

The authors declare no conflict of interest.

## Author Contributions

J.S. and L.Z. contributed equally to this work. X.W. designed the experiments, J.S., L.Z., Y.L., J.T., and H.Z. performed the experiments. J.S., L.Z., Y.L., T.Z., W.W., and L.L. analyzed the data. J.S., L.Z., and X.W. wrote the manuscript with help from all authors. X.W. and S.B. revised the manuscript. X.W. and S.B. supervised the work. All of the authors reviewed and approved the manuscript.

## Supporting information



Supporting Information

## Data Availability

The data that support the findings of this study are available on request from the corresponding author. The single‐cell RNA‐seq data from this study have been submitted to the National Center for Biotechnology Information GEO database and GEO number is GSE302023.
