# Supplementary material for: Ex Vivo Spatiotemporal Characterization of Spermatogenesis in Mouse Testicular Organoids
Source: Adv Sci (Weinh). 2025 Oct 31;13(4):e12670. doi: 10.1002/advs.202512670 (PMC12822388; doi:10.1002/advs.202512670)
Supplement: Supplementary file 1 — Supporting Information [file ADVS-13-e12670-s001.docx]

***Ex Vivo* Spatiotemporal Characterization of Spermatogenesis in Mouse Testicular Organoids**

Jiachen Sun, Lifa Zhu, Yutong Li, Jinyan Tang, Tao Zhang, Hengjia Zhang, Wanchu Wang, Lufan Li, Shuhui Bian, Xin Wu^*^

**Supplementary Figures**

**
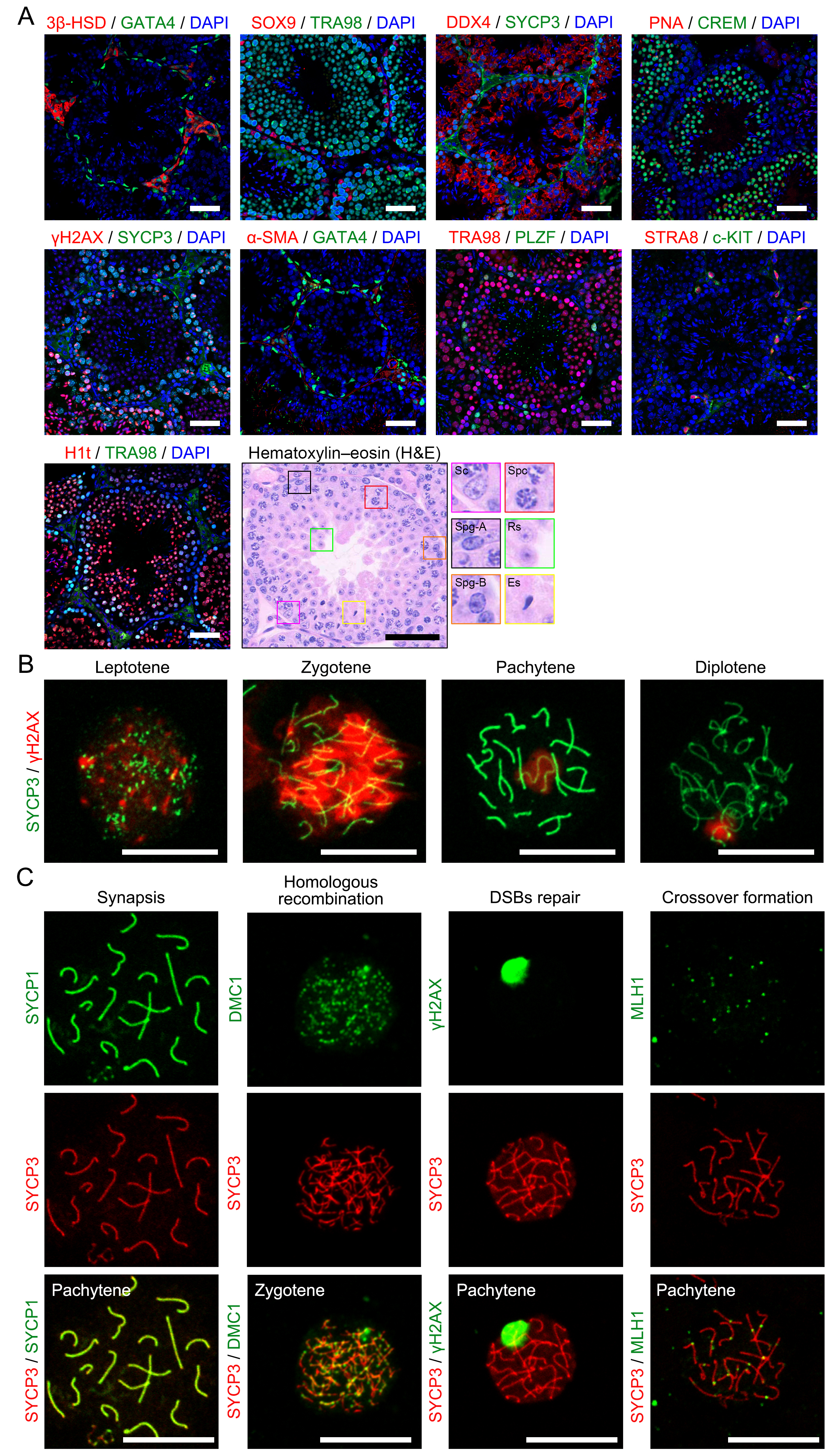
**

**Figure S1. Antibody immunofluorescence and Hematoxylin‒eosin (H&E) staining of mouse testis sections and spermatocyte chromosome spreads.**

(A) Immunofluorescence staining of adult mouse testis sections (day 56) and Hematoxylin‒eosin (H&E) staining of mouse testis section (day 28), Sertoli cells (Sc, purple box), type A spermatogonia (Spg-A, black box), type B spermatogonia (Spg-B, orange box), spermatocytes (Spc, red box), round spermatids (Rs, green box) and elongated spermatids (Es, yellow box) are indicated in the representative organoid sections. Scale bar, 50 µm.

(B) Immunofluorescence staining of SYCP3 (green) and γH2AX (red) in testicular meiotic spermatocytes at different stages of prophase I (leptotene, zygotene, pachytene, and diplotene), showing chromosome distribution patterns and colocalization of the marker proteins. Scale bar, 20 μm.

(C) Immunofluorescence images of chromosome spreads from testicular meiotic spermatocytes (day 21), co-stained for SYCP1 (green), DMC1 (green), γH2AX (green), MLH1 (green), and SYCP3 (red). Scale bar, 20 μm.

**
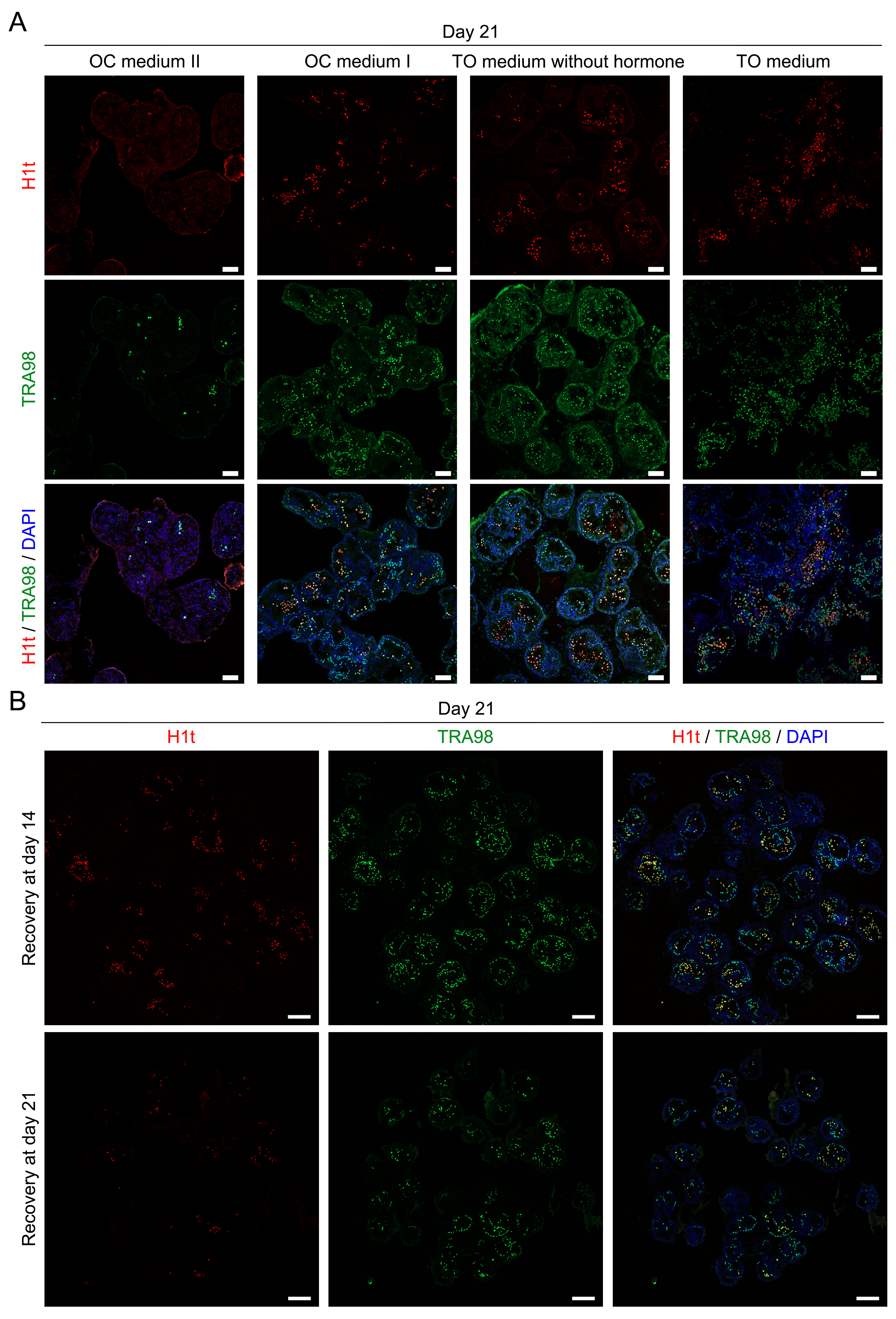
**

**Figure S2. Effects of culture medium components and ECM-removal time points on germ cell development in testicular organoids.**

1. From top to bottom, H1t (red) labels mid-late pachytene spermatocytes and post-meiotic germ cells, while TRA98 (green) labels germ cells. DAPI (blue) is used to stain cell nuclei; and from left to right, OC Medium II, OC Medium I, and TO Medium without hormones, compared to TO Medium supplemented with hormones and antioxidants. The tables in the Supplementary Information (Tables S1–S3) show the differences between the various media components. Scale bar, 100 µm.
2. Immunostaining of testicular organoids removed from the ECM on day 21 (bottom) reveals a significant loss of germ cells compared to testicular organoids removed from the ECM on day 14 (top). H1t (red) labels mid- to late-pachytene spermatocytes and post-meiotic germ cells, while TRA98 (green) labels germ cells. DAPI (blue) was used to stain nuclei. Scale bar, 200 µm.


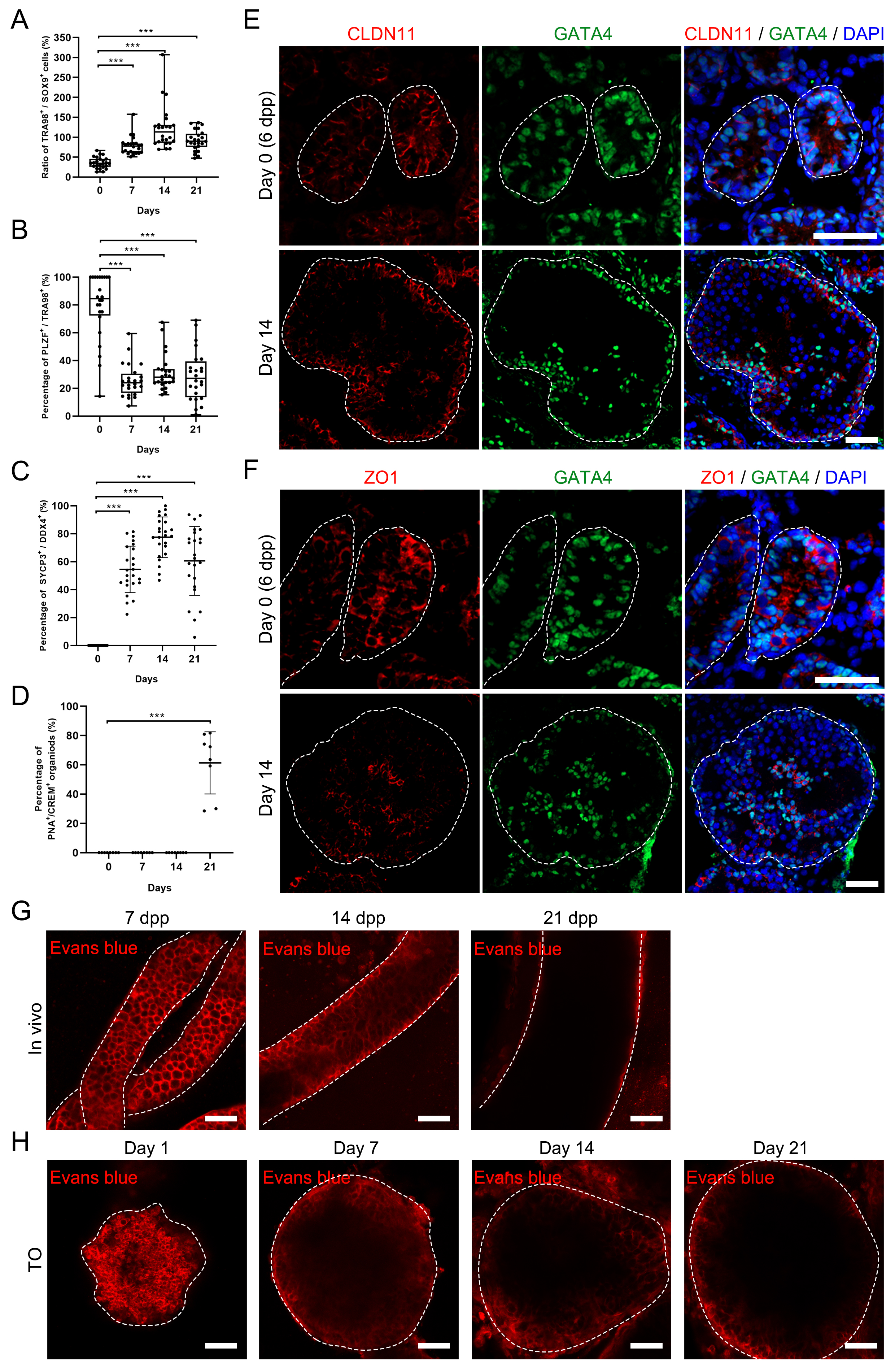


**Figure S3. Germ cell development and blood‒testis barrier formation in TOs.**

(A–D) Quantification of cell populations in TOs at the indicated time points (days 0, 7, 14 and 21). Ratio of TRA98^+^ to SOX9^+^ cells, n = 24 (A). Percentage of PLZF⁺ cells within the TRA98⁺germ cell population, n = 24 (B). Percentage of SYCP3⁺ cells within the DDX4⁺ cell population, n = 24 (C). Percentage of tubules containing round spermatids (RS) over 21 days of culture, n = 8 (D). The data are presented as the means ± SDs (C and D) or median with interquartile range (A and B). Each dot represents an individual organoid. Statistical significance was determined by one-way Analysis of Variance (ANOVA) with Tukey’s multiple comparisons test (C and D). For the data sets in which at least one group did not follow a normal distribution (A and B), Mann-Whitney U test was used. *** indicates *P* < 0.001.

(E–F) Representative immunofluorescence images of testicular organoids on day 0 (6 dpp mouse testis) and day 14. Coimmunostaining for CLDN11 (red, tight junction protein) and GATA4 (green, somatic cell marker) revealed the formation of organized seminiferous tubule-like structures with proper cell polarization by day 14 (E). Coimmunostaining for ZO1 (red, tight junction protein) and GATA4 (green) confirmed the establishment of functional blood‒testis barrier components (F). The white dashed lines outline organoid boundaries. Nuclei were counterstained with DAPI (blue). Scale bar, 50 μm.

(G–H) In vivo testicular seminiferous tubules and in vitro testicular organoids were stained with 0.4% Evans blue. The white dashed line outlines the boundary between the seminiferous tubules and organoids. Scale bar, 50 µm.


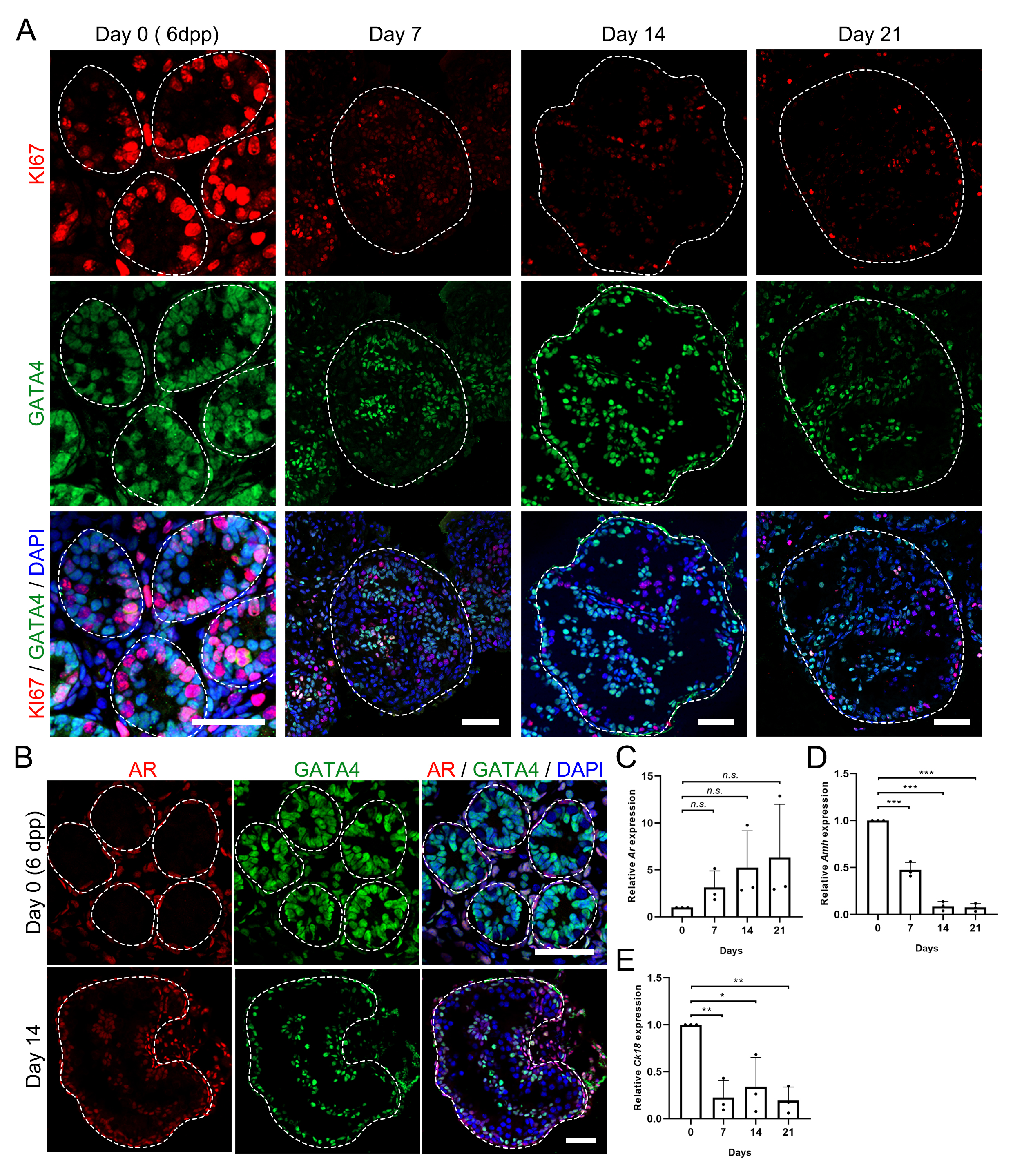


**Figure S4. Characterization of Sertoli cell maturation in vitro.**

1. Immunofluorescence staining of testicular organoids for KI67 (red) and GATA4 (green) on days 0 (6 dpp mouse testis), 7, 14 and 21 of culture. Nuclei were counterstained with DAPI (blue). Scale bar, 50 μm. The dashed white lines indicate the boundaries of the organoids.
2. Immunofluorescence staining of testicular organoids for AR (red) and GATA4 (green) on days 0 (6 dpp mouse testis) and 14 of culture. Nuclei were counterstained with DAPI (blue). Scale bar, 50 μm. The dashed white lines indicate the boundaries of the organoids.

(C–E) qRT‒PCR analysis of *Ar* (C), *Amh* (D), and *Ck18* (E) expression in testicular organoids on days 0, 7, 14, and 21 of culture. Relative gene expression levels were normalized to that of *Actb* (*β-Actin*), which was used as a reference gene. The data are presented as the means ± SDs (biologically independent samples, n=3). Statistical significance was determined by one-way Analysis of Variance (ANOVA) with Tukey’s multiple comparisons test. *n.s.*: not significant, * indicates *P* < 0.05, ** indicates *P* < 0.01, *** indicates *P* < 0.001.


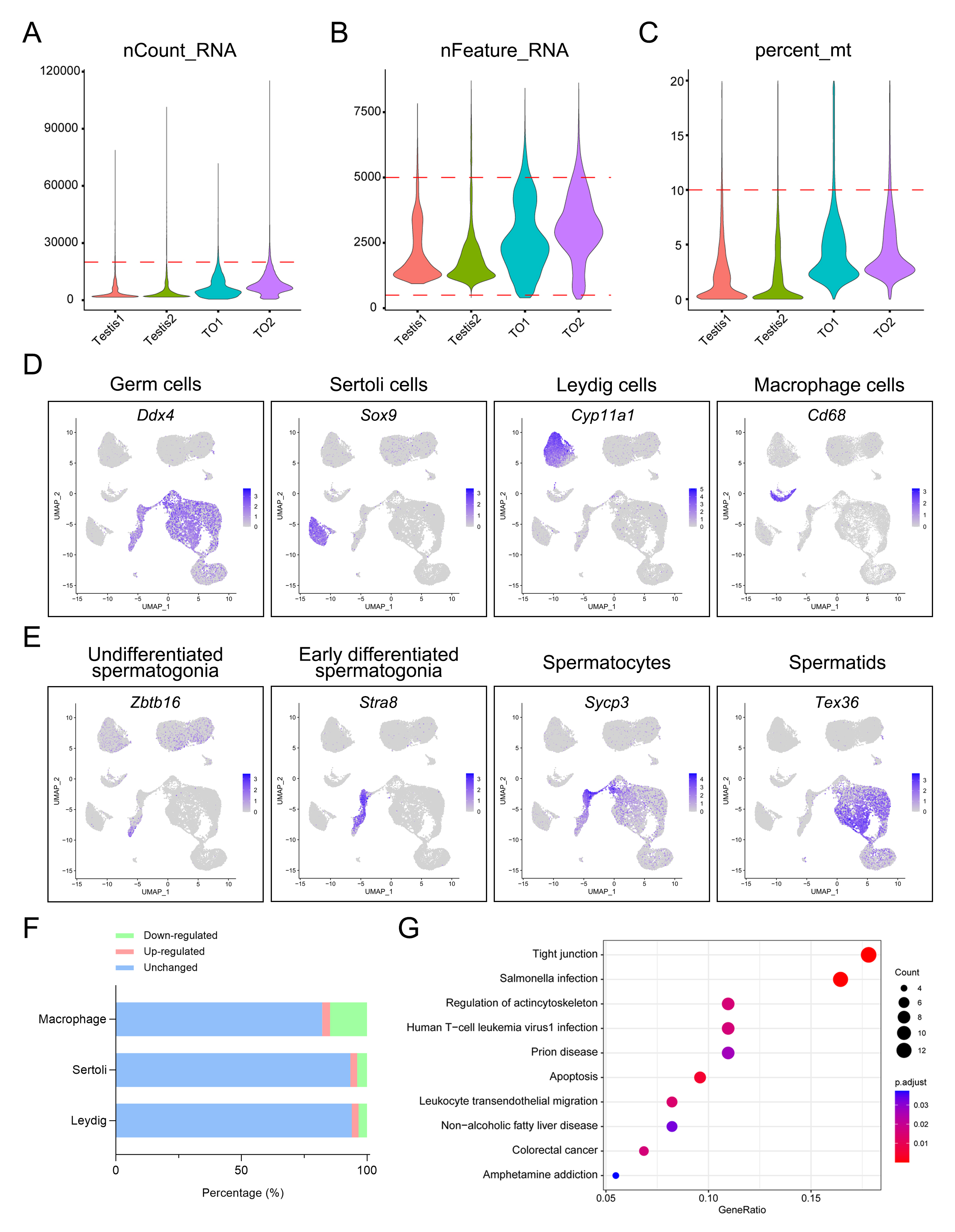


**Figure S5. Single-cell transcriptomic profiling of testis and testicular organoids.**

(A–C) Quality control (QC) metrics for single-cell RNA sequencing. Violin plots displaying (A) the number of unique molecular identifiers (UMIs) (nCounts_RNA), (B) the number of detected features (genes) (nFeature_RNA), and (C) the percentage of mitochondrial reads (percent_mt) per cell across two biological replicates of in vivo testes (Testis1, Testis2) and two TO samples (TO1, TO2). The red dashed lines represent the filtering thresholds used (nCounts_RNA < 20,000; 500 < nFeature_RNA < 5,000; percent_mt < 10%).

(D) Uniform manifold approximation and projection (UMAP) plots showing the expression of canonical marker genes for major testicular cell populations: *Ddx4* for germ cells, *Sox9* for Sertoli cells, *Cyp11a1* for Leydig cells, and *Cd68* for macrophage cells. The color intensity was correlated with the normalized gene expression levels.

(E) UMAP plots illustrating the expression of marker genes for distinct stages of germ cell development: *Zbtb16* for undifferentiated spermatogonia, *Stra8* for early differentiated spermatogonia, *Sycp3* for spermatocytes, and *Tex36* for spermatids. The color intensity was correlated with the normalized gene expression levels.

(F) Bar chart depicting the percentage of downregulated (green), upregulated (red), and unchanged (blue) genes in macrophages, Sertoli cells, and Leydig cells from TOs compared with in vivo testes.

(G) Dot plot showing the top enriched KEGG pathways in Sertoli cells for differentially expressed genes between testes and TOs. The dot size represents the number of genes enriched in the pathway, and the dot color represents the adjusted p value.


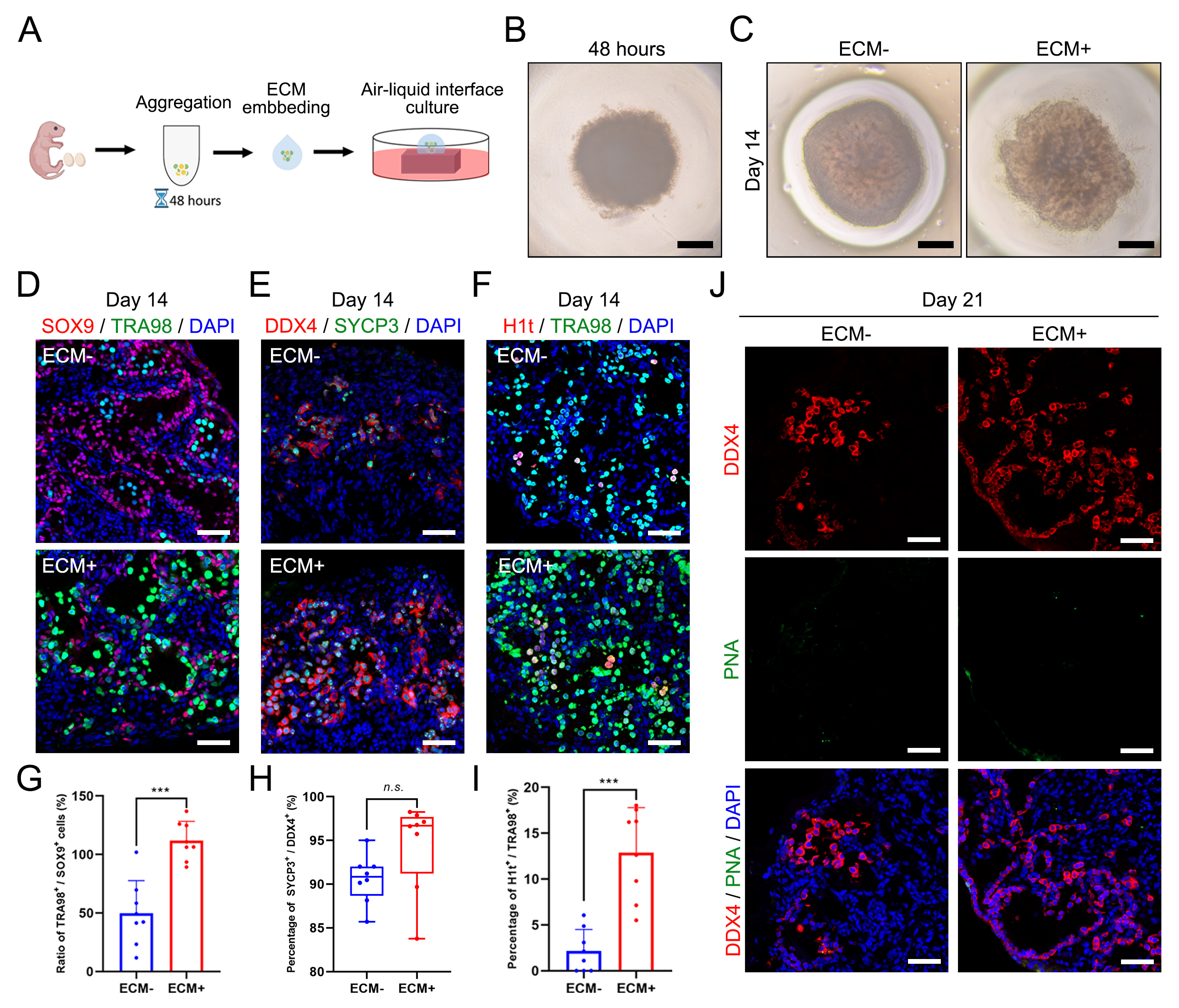


**Figure S6. ECM embedding promotes germ cell maintenance and meiosis in testicular organoids.**

(A) Schematic illustration of the workflow for generating testicular organoids. Single cells isolated from pup testes were allowed to aggregate for 48 hours to form initial spheroids (200,000 cells per spheroid). These aggregates were then cultured with or without extracellular matrix (ECM).

1. A representative bright-field image of a spheroid after 48 hours of aggregation is shown. Scale bar, 100 µm.
2. Representative bright-field images of organoids cultured with (ECM+) or without ECM (ECM-) on day 14. Scale bar, 100 μm.

(D–F) Immunofluorescence analysis of testicular organoids on day 14. Confocal images showing Sertoli cells (SOX9, red) and germ cells (TRA98, green) (D). Confocal images showing germ cells (DDX4, red) and meiotic germ cells (SYCP3, green) (E). Confocal images showing mid‒late pachytene spermatocytes (H1t, red) and germ cells (TRA98, green) (F). Nuclei were counterstained with DAPI (blue). Scale bar, 50 μm.

(G–I) Quantification of the ratio of TRA98^+^ cells to SOX9^+^ cells, n = 8 (G), the percentage of SYCP3^+^/DDX4^+^ cells, n = 8 (H), and the percentage of H1t^+^/TRA98^+^ cells, n = 8 (I) in organoids cultured with or without ECM on day 14. The data are presented as the means ± SDs (G and I) or median with interquartile range (H) from at least 3 independent experiments per group. Each dot represents an individual organoid. Statistical significance was determined by unpaired Student’s t-test (G and I). For the data sets in which at least one group did not follow a normal distribution (H), Mann-Whitney U test was used. *n.s.*: not significant, *** indicates *P* < 0.001.

(J) Immunofluorescence staining of day 21 organoids for the germ cell marker DDX4 (red) and the acrosomal marker peanut agglutinin (PNA, green). Nuclei were counterstained with DAPI (blue). Representative images show individual channels and merged images for the ECM- (left column) and ECM+ (right column) conditions. Scale bar, 50 µm.


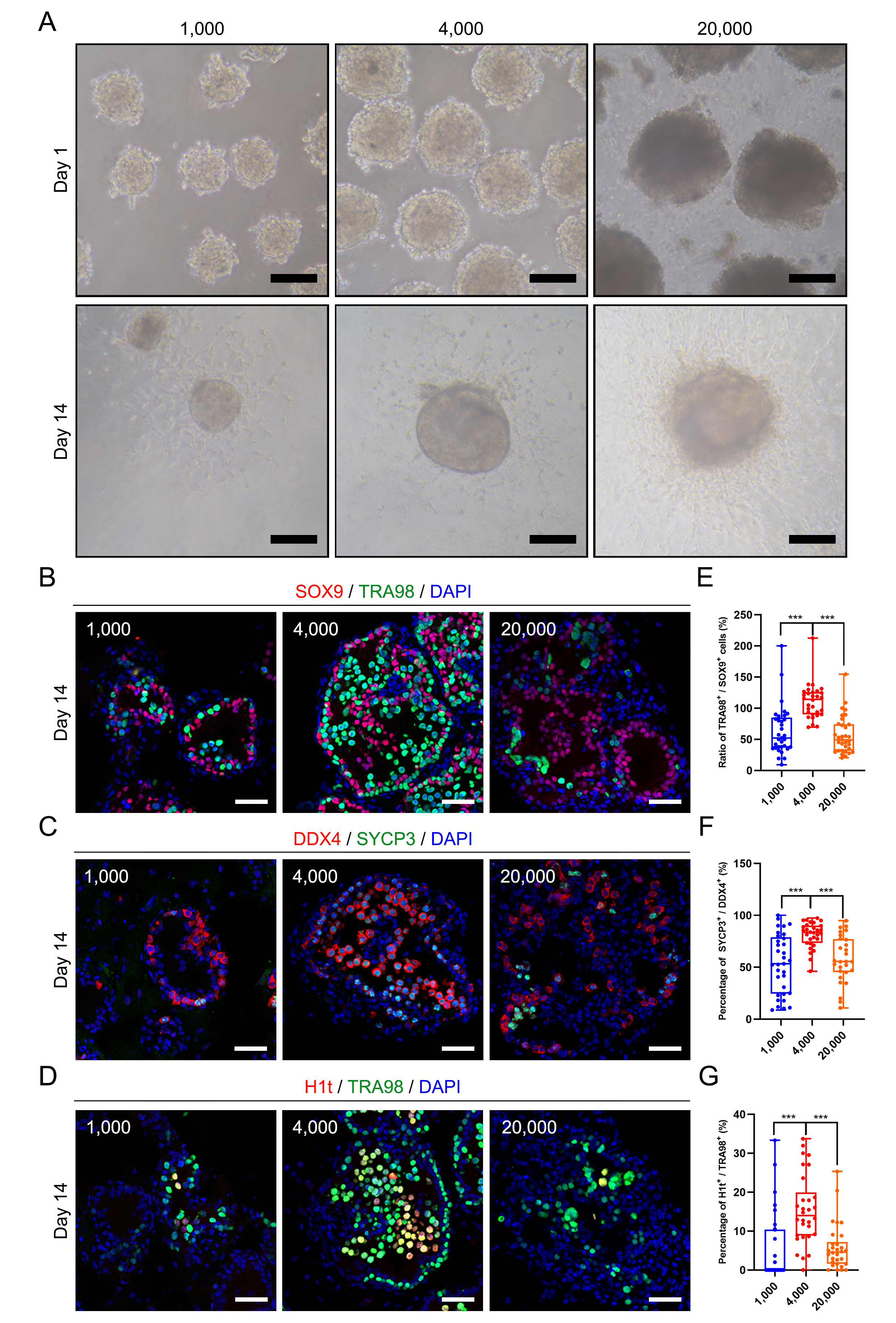


**Figure S7. Effects of initial cell seeding density on TO morphogenesis and spermatogenesis.**

1. Bright-field images showing the morphological development of testicular organoids generated from different initial cell seeding densities (1,000, 4,000, and 20,000 cells per organoid) on days 1 and 14 of culture. Scale bar, 100 µm.

(B–D) Immunofluorescence staining of testicular organoids on day 14. Staining for SOX9 (red) and TRA98 (green) (B). Staining for DDX4 (red) and SYCP3 (green) (C). Staining for H1t (red) and TRA98 (green) (D). Nuclei were counterstained with DAPI (blue). Scale bar, 50 μm.

(E–G) Quantification of the ratio of TRA98⁺/SOX9⁺ cells, n = 30–33 (E), the percentage of SYCP3⁺/DDX4⁺ cells, n = 28–33 (F), and the percentage of H1t⁺/TRA98⁺ cells, n = 30 (G) in testicular organoids derived from different initial cell numbers. The data are presented as the median with interquartile range. Statistical significance was determined using Mann-Whitney U test. Each dot represents an individual organoid. *** indicates *P* < 0.001.


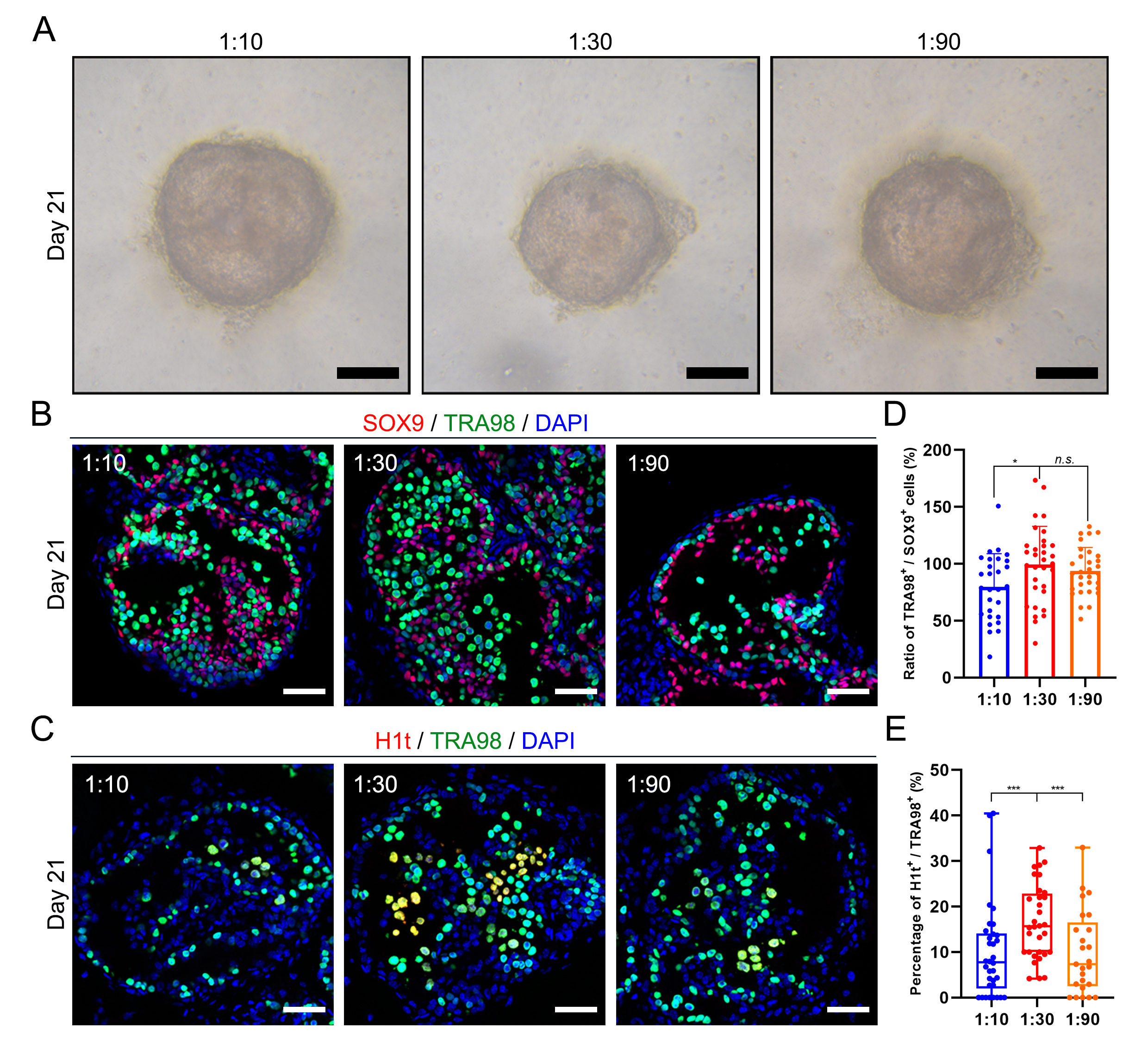


**Figure S8. Effect of initial germ cell abundance on germ cell development in testicular organoids.**

1. Representative bright-field images of TOs on day 21 formed with different initial proportions of germ cells (1:10, 1:30, and 1:90). Scale bar, 100 μm.

(B–C) Immunofluorescence staining of testicular organoids with different initial germ cell ratios on day 21. Staining for SOX9 (red) and TRA98 (green) (B). H1t (red) and TRA98 (green) (C) were stained. Nuclei were counterstained with DAPI (blue). Scale bar, 50 μm.

(D–E) Quantification of the ratio of TRA98⁺/SOX9⁺ cells, n = 28–33 (D) and the percentage of H1t⁺/TRA98⁺ cells, n = 25–34 (E) in testicular organoids on day 21. The data are presented as the means ± SDs (D) or median with interquartile range (E). Each dot represents an individual organoid. Statistical significance was assessed using one-way ANOVA followed by Tukey’s multiple comparisons test (D). For the data sets in which at least one group did not follow a normal distribution (E), Mann-Whitney U test was used. *n.s.*: not significant, * indicates *P* < 0.05, and *** indicates *P* < 0.001.

**Supplementary Tables**

**Table S1. Organ culture medium I (100 mL)**

| Reagent | Manufacturer | Catalog number | Final volume  (100 mL) | Final conc. |
| --- | --- | --- | --- | --- |
| MEMα | Gibco | 12561049 | 89 mL | Basal medium |
| KnockOut serum replacement | Gibco | 10828028 | 10 mL | 10% |
| Penicillin‒Streptomycin | Gibco | 15140122 | 1 mL | Penicillin: 50 units/mL  Streptomycin: 50 μg/mL |

**Table S2. Organ culture medium II (100 mL)**

| Reagent | Manufacturer | Catalog number | Final volume  (100 mL) | Final conc. |
| --- | --- | --- | --- | --- |
| MEMα | Gibco | 12561049 | 99 mL | Basal medium |
| AlbuMax I | Gibco | 11020021 | 4 g | 40 mg/mL |
| Penicillin‒Streptomycin | Gibco | 15140122 | 1 mL | Penicillin: 50 units/mL  Streptomycin: 50 μg/mL |

**Table S3. Composition of testicular organoid culture medium (100 mL)**

| Reagent | Manufacturer | Catalog number | Stock solution | Final volume  (100 mL) | Final conc. |
| --- | --- | --- | --- | --- | --- |
| MEMα | Gibco | 12561049 | N/A | 88 mL | Basal medium |
| KnockOut serum replacement | Gibco | 10828028 | N/A | 10 mL | 10% |
| Penicillin‒Streptomycin | Gibco | 15140122 | N/A | 1 mL | Penicillin: 50 units/mL  Streptomycin: 50 μg/mL |
| L-Ascorbic acid 2-Glucoside | TCI | G0394 | 100 mM | 500 μL | 0.5 mM |
| DL-alpha-Tocopherol acetate | Sigma | T3376 | 1 M | 50 μL | 0.5 mM |
| L-Glutathione | Sigma | G6013 | 50 mg/mL | 308 μL | 0.5 mM |
| Testosterone | Merck | T-037 | 1 mM | 100 μL | 1 μM |
| Follicle-stimulating hormone | Sigma | F4021 | 1 μg/mL | 100 μL | 1 ng/mL |

**Table S4. Primers for qRT-PCR**

| Gene | Forward | Reverse |
| --- | --- | --- |
| *Actin* | 5’-CCGTAAAGACCTCTATGCC-3’ | 5’-CTCAGTAACAGTCCGCCTA-3’ |
| *Amh* | 5’-TTGGTGCTAACCGTGGACTTC-3’ | 5’-CGGGAATCAGAGCCAAATAGAAA-3’ |
| *Ck18* | 5’-TCAAGATCATCGAAGACCTGAGG-3’ | 5’-GCGCATGGCTAGTTCTGTC-3’ |
| *Ar* | 5’-TCCAAGACCTATCGAGGAGCG-3’ | 5’-GTGGGCTTGAGGAGAACCAT-3’ |

**Table S5. Primary antibodies for Immunofluorescence and Spermatocyte Nuclear Spreading Analyses**

| Antigen | Company | Catalog nº | Species |
| --- | --- | --- | --- |
| GATA4 | Santa Cruz Biotechnology | sc-1237 | Goat |
| 3β-HSD | Proteintech | 15516-1-AP | Rabbit |
| α-SMA | Proteintech | 14395-1-AP | Rabbit |
| ZO-1 | Proteintech | 21773-1-AP | Rabbit |
| Claudin-11 | Affinity | AF5364 | Rabbit |
| SOX9 | Millipore | AB5535 | Rabbit |
| TRA98 | Abcam | ab82527 | Rat |
| DDX4 | Abcam | ab13840 | Rabbit |
| PLZF | R&D Systems | AF2944 | Goat |
| STRA8 | Abcam | ab308124 | Rabbit |
| c-KIT | R&D Systems | AF1356 | Goat |
| SYCP3 | Abcam | ab97672 | Mouse |
| γH2AX | Abcam | ab11174 | Rabbit |
| H1t | ABclonal | A18597 | Rabbit |
| CREM | Proteintech | 12131-1-AP | Rabbit |
| Caspase-3 | Proteintech | 25128-1-AP | Rabbit |
| KI67 | Abcam | ab15580 | Rabbit |
| AR | Abcam | ab133273 | Rabbit |
| SYCP1 | Abcam | ab15090 | Rabbit |
| DMC1 | Proteintech | 13714-1-AP | Rabbit |
| MLH1 | Proteintech | 11697-1-AP | Rabbit |
